# Protocol to identify small molecules promoting rat and mouse cardiomyocyte proliferation based on the FUCCI and MADM reporters

**DOI:** 10.1016/j.xpro.2022.101903

**Published:** 2022-12-05

**Authors:** Lixia Zheng, Zihao Wang, Jianyong Du, Xiaojun Zhu, Jing-Wei Xiong

**Affiliations:** 1 Beijing Key Laboratory of Cardiometabolic Molecular Medicine, Institute of Molecular Medicine, College of Future Technology, and State Key Laboratory of Natural and Biomimetic Drugs, Peking University, Beijing 100871, China; 2 PKU-Nanjing Institute of Translational Medicine, Nanjing 211800, China; 3 School of Health and Life Sciences, University of Health and Rehabilitation Sciences, Qingdao 266114, China

**Keywords:** Cell Biology, Cell isolation, Cell-based Assays, High Throughput Screening, Microscopy

## Abstract

Discovery of small molecules promoting cardiomyocyte proliferation is important for heart regeneration and related heart disease. Here, we describe a protocol to isolate neonatal rat and mouse cardiomyocytes, infect cardiomyocytes with Tnnt2-mAG-hGeminin (1/110) or Tnnt2-Cre adenovirus, and identify small molecules that promote cardiomyocyte proliferation by high-content microscopy. This protocol can be modified to investigate other pro-proliferation factors in cardiomyocytes and other cell types.

For complete details on the use and execution of this protocol, please refer to Du et al. (2022).^1^

## Highlights

• Isolating and culturing neonatal mouse and rat ventricular myocytes
• Performing chemical screen for pro-proliferation small molecules using FUCCI
• Identifying small molecules enabling cardiomyocyte cytokinesis using MADM

## Abstract (editor highlights)

Publisher’s note: Undertaking any experimental protocol requires adherence to local institutional guidelines for laboratory safety and ethics.

## Graphical abstract

**Figure:**
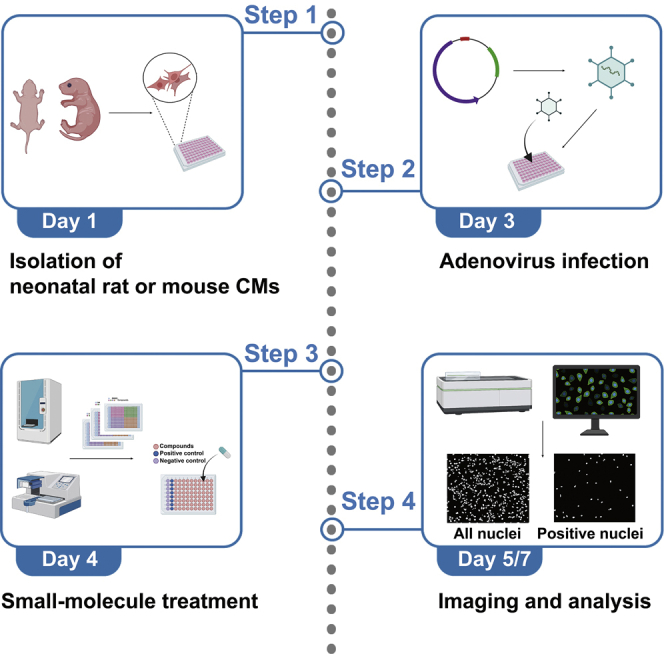

## Before you begin

### Institutional permissions

Neonatal (postnatal day 3, P3) wild-type Sprague-Dawley (SD) rats used for this work were purchased from Vital River Laboratory Animal Technology Co., Ltd (Beijing, China). MADM-ML-11^TG^ and MADM-ML-11^GT^ mouse lines were kindly provided by Dr. Chong Liu at the College of Medicine, Zhejiang University (Hangzhou, China).^2^ All procedures involving experimental mice and rats were approved by the Institutional Animal Care and Use Committee at Peking University, Beijing, China.

### Obtain the FUCCI reporter plasmid

**Timing: 1 week**

**Figure 1:**
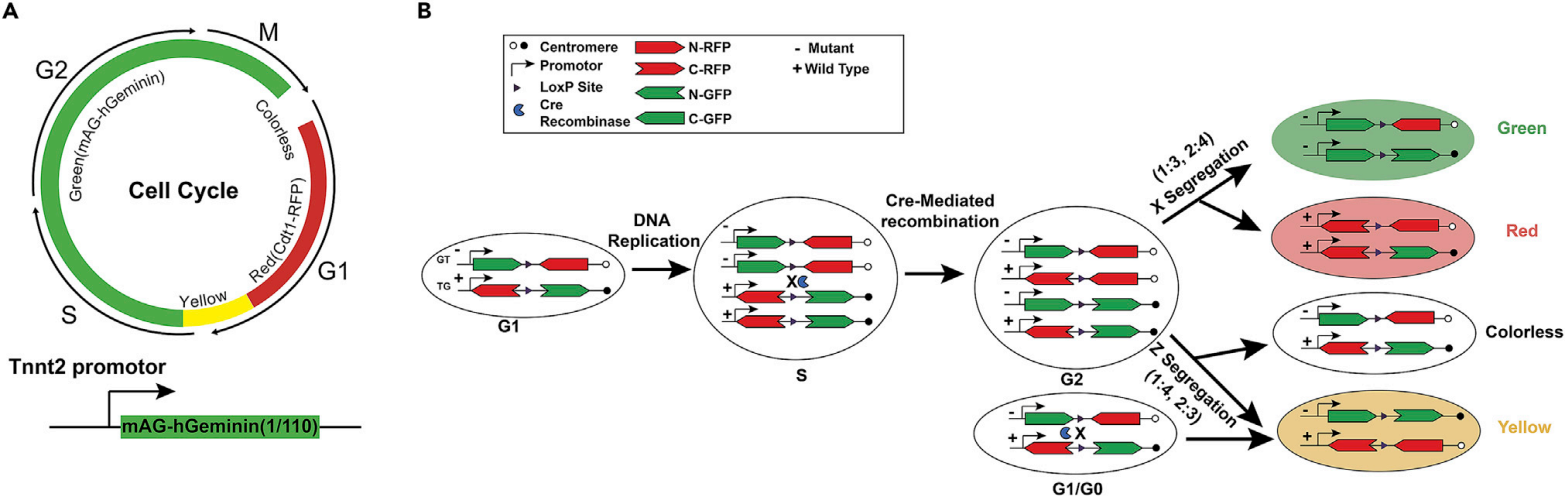
Schematic of the FUCCI and MADM system (A) The FUCCI system consists of two parts: RFP red fluorescent protein fused with 30–120 amino-acid residues of human Cdt1 (hCdt1) protein expressed in G1 phase; and mAG green fluorescent protein fused with amino-acid residues at sites 1–110 of human Geminin protein (hGeminin) expressed in S/G2/M phase. Cells expressing FUCCI exhibited red fluorescence in the G1 phase, green fluorescence in the S/G2/M phase, and dual fluorescence in the G1/S transition phase. (B) The MADM system consists of two knock-in genes, which are chimeric genes consisting of the N- and C-terminal of two different fluorescent proteins, such as RFP and GFP. The chimeric N-RFP/C-GFP or N-GFP/C-RFP alleles produce two non-functional proteins before Cre-mediated recombination. During the S phase of mitosis, sister chromatids replicate and pair. If inter-chromosomal recombination occurs, functional GFP and/or RFP are produced. Green and red daughter cells or yellow and colorless daughter cells are generated after mitosis.

The FUCCI system consists of two parts: RFP red fluorescent protein fused with 30–120 amino-acid residues of human Cdt1 (hCdt1) protein expressed in G1 phase; and mAG green fluorescent protein fused with amino-acid residues at sites 1–110 of human Geminin protein (hGeminin) expressed in S/G2/M phase. Therefore, cells expressing FUCCI exhibited red fluorescence in the G1 phase, green fluorescence in the S/G2/M phase, and dual fluorescence in the G1/S transition phase^3^^,^^4^ (Figure 1A). To simplify the screening process, we used only one part of the FUCCI system: mAG-hGeminin to label the cells entering S/G2/M phase. This study was designed to identify small molecules that significantly promote the entry of cardiomyocytes into S/G2/M phase by infecting cardiomyocyte-specific Tnnt2-mAG-hGeminin adenovirus into neonatal rat ventricular cardiomyocytes and subsequently applying chemical treatment.

1. Double-digest the H202 vector plasmid with restriction enzymes XbaI and AgeI-HF in the Cutsmart buffer system (refer to Table 1 for the restriction enzyme digestion system).

**Table 1:** Plasmid double-digestion system

Reagent	Amount
DNA	1 μg
XbaI	0.5 μL
AgeI-HF	0.5 μL
10× Cutsmart Buffer	2 μL
Deionized H_2_O	Add to 20 μL

**Figure 2:**
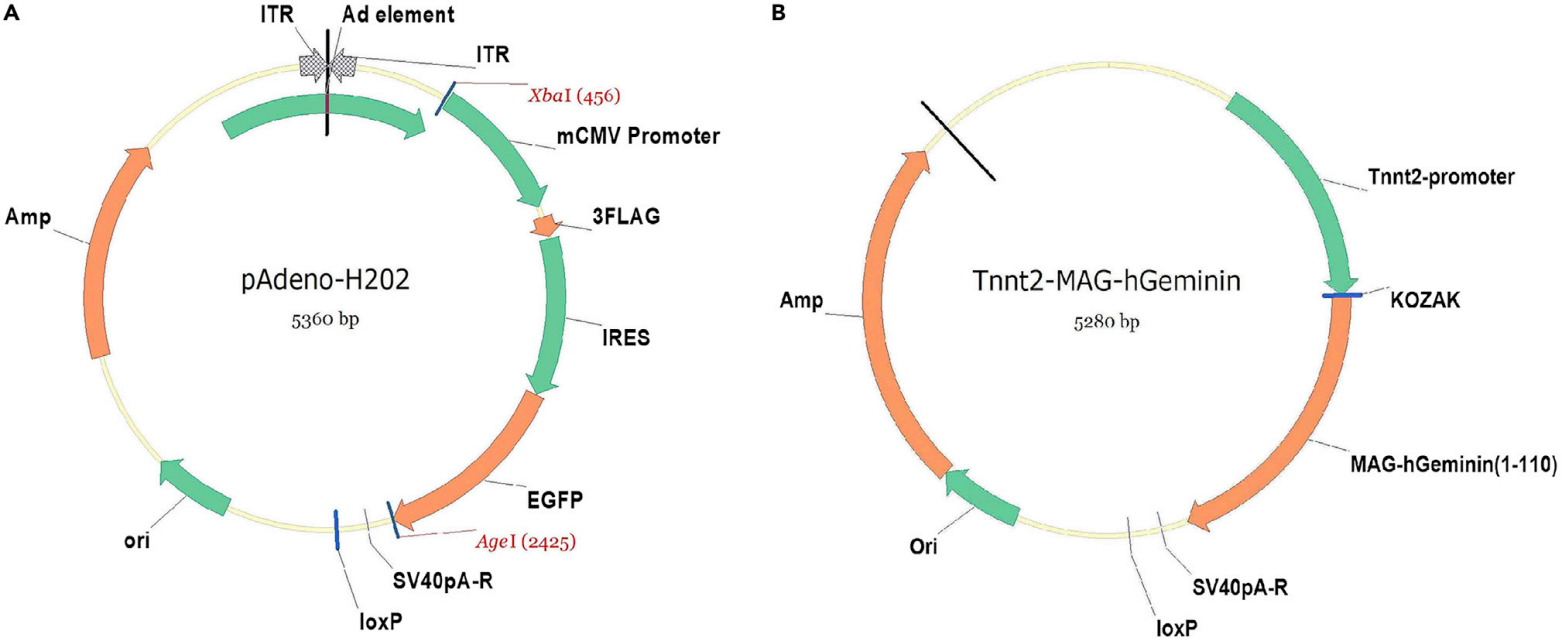
Maps of the H202 pAdeno-MCMV-3Flag-IRES2-EGFP vector plasmid and pAdeno-Tnnt2-mAG-hGeminin(1/110) plasmids (A) The H202 pAdeno-MCMV-3Flag-IRES2-EGFP vector plasmid contains XbaI and AgeI-HF restriction enzyme cut sites. Double-digest the H202 vector plasmid with restriction enzymes XbaI and AgeI-HF to obtain the vector fragment. (B) mAG-hGeminin (1/110) is driven by the cardiomyocyte-specific Tnnt2 promoter and cloned into H202 vector.

***Note:*** The pAdeno-MCMV-3Flag-IRES2-EGFP vector plasmid (H202) was purchased from OBiO Technology Corp., Ltd., Shanghai (Figure 2A).

**Figure 3:**
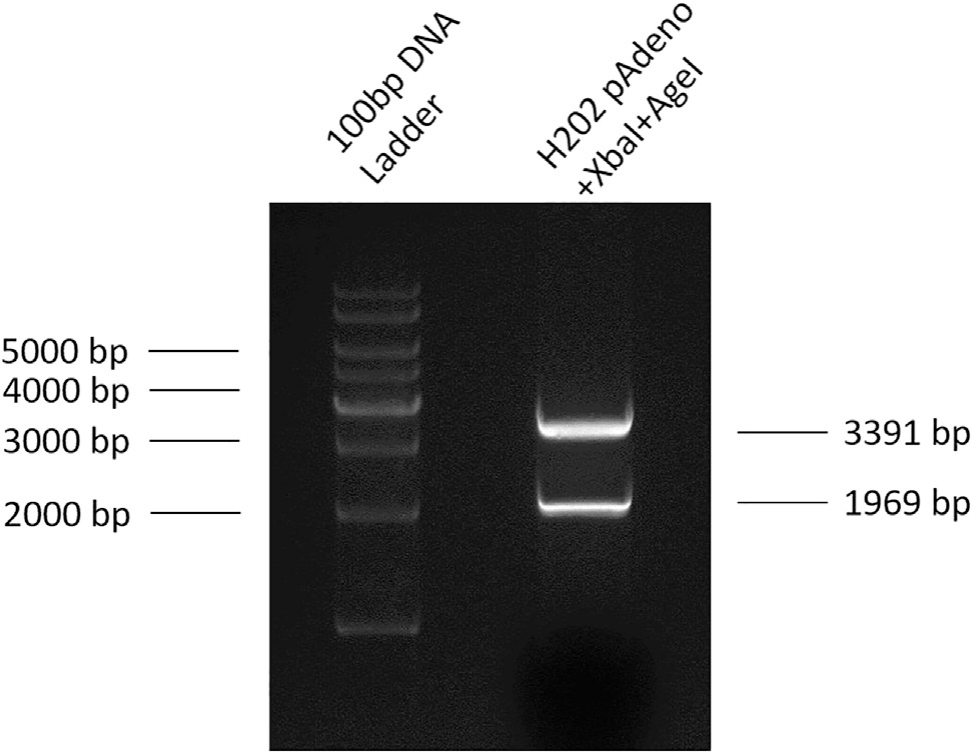
Result of agarose gel electrophoresis after the H202 vector plasmid is double digested with restriction enzymes XbaI and AgeI-HF

***Note:*** Keep the enzyme on ice when not in the freezer and the enzyme should be the last component added to the reaction.

2. Separate the vector and DNA fragments by gel electrophoresis, and then collect the vector fragments (Figure 3).

3. Design PCR primers with overlapping sequences for the Tnnt2 promoter, mAG-hGeminin (1/110) cDNA, and the H202 vector, the length of overlapping sequences is 20 bp.

***Note:*** DNA plasmids containing either the Tnnt2 promoter or mAG-hGeminin (1/110) sequences are available from our laboratory (Figure 2B) (seen in Data S1).

4. Carry out PCR reactions using the primers (see Table 2 for primers, Tables 3 and 4 for the PCR system and program settings).

**Table 2:** Primer sequences for plasmid construction and mouse genotyping

**pAdeno-Tnnt2-mAG-hGeminin(1/110) plasmid**		
Tnnt2 promoter forward primer	TTATTATAGTCAGNTCTAGATGTAGTTAATGATTAACCCGC	
Tnnt2 promoter reverse primer	ATCACGCTCACCATGGTGGCCTATAGTGAGTCGTATTAAG	
mAG-hGeminin (1/110) forward primer	CTTAATACGACTCACTATAGGCCACCATGGTGAGCGTGAT	
mAG-hGeminin (1/110) reverse primer	GATATCGAATTACCGGTTTACAGCGCCTTTCTCCGTTTTT	

**pAdeno-Tnnt2-mAG-hGeminin(1/110) plasmid confirmed primer**		

Forward primer	GGGCCGCGGGGACTTTGACC	
Reverse primer	GAAATTTGTGATGCTATTGC	

**pAdeno-Tnnt2-Cre plasmid**		
Tnnt2 promoter forward primer	TTATTATAGTCAGNTCTAGATGTAGTTAATGATTAACCCGC	
Tnnt2 promoter reverse primer	AGTAAATTGGCCATGGTGGCCTATAGTGAGTCGTATTAAG	
Cre forward primer	ACGACTCACTATAGGCCACCATGGCCAATTTACTGACCGT	
Cre reverse primer	CTTGATATCGAATTACCGGTCTAATCGCCATCTTCCAGCA	

**pAdeno-Tnnt2-Cre plasmid confirmed primer**		
Forward primer	TTATTATAGTCAGNTCTAGATGTAGTTAATGATTAACCCG	
Reverse primer	TTGATTATCGATAAGCTTGATATCGAATTACCGGTCTAAT	

**Primers used for MADM-ML-11**^**TG**^**and MADM-ML-11**^**GT**^**mice genotyping**		
MADM-F1	CGTGCTGGTTATTGTGCTGT	MADM-ML-11^TG^:210 bp; MADM-ML-11^GT^:500 bp
MADM-R1	TTCGGAGATCCATAACTTCG	
MADM-F2	AAGGTTCTTATCCCCTGGAAG	Wild Type: 650 bp
MADM-R2	CCTTCAGCTGCCCACTCTAC

**Table 3:** PCR reaction system

Reagent	Amount
PrimeSTAR Max Premix (2×)	25 μL
Forward primer	10 pmol
Reverse primer	10 pmol
Template plasmid	<200 ng
Deionized H_2_O	Up to 50 μL

**Table 4:** PCR program for amplifying Tnnt2 and Cre fragments

Step	Temperature	Time	Cycles
Denaturation	98°C	10 s	30 cycles
Annealing	55°C	15 s	
Extension	72°C	1 min	
Hold	10°C	hold
5. Separate the PCR product fragments by agarose gel electrophoresis.
6. Use the Tiangen Universal DNA Purification Kit to recover the target PCR products.
7. Clone the amplified Tnnt2 promoter and mAG-hGeminin (1/110) into the H202 vector using the NEBuilder HiFi DNA Assembly Reaction System (Table 5).

**Table 5:** NEBuilder HiFi DNA assembly reaction system

Reagent	Amount
Recommended DNA molar ratio	vector: insert = 1:2
Total amount of fragments	0.03–0.2 pmol
NEBuilder master mix	10 μL
Deionized H_2_O	Add to 20 μL

***Note:*** Pre-chill reagents on ice and keep on ice during the whole process.

8. Incubate the prepared assembly system in a thermal cycler and maintain at 50°C for 15 min.
9. Thaw a 50 μL tube of DH5α-competent cells on ice for 5 min.
10. Add 2 μL of the assembly reaction, gently flick the tube to mix 4–5 times.
11. Incubate on ice for 30 min.
12. Heat shock at 42°C for 90 s.
13. Place back on ice for 10 min.
14. Add 950 μL of room temperature (18°C–26°C) LB medium. Incubate in a shaker at 200 rpm at 37°C for 1 h.
15. Warm LB agar plates containing Ampicillin at 37°C for 15 min.
16. Thoroughly mix the cells by flicking the tube and inversion, then spread 100 μL bacteria onto each plate.
17. Incubate the plates at 37°C for 12 h.
18. Select single-clone colonies and inoculate in LB medium at 37°C, 220 rpm for 16 h.
  a. Extract and identify plasmids using agarose gel electrophoresis.
  b. Confirm FUCCI reporter plasmid by sequencing with forward and reverse primers in Table 2.

### Virus packaging and identification

**Timing: 3 weeks**

OBiO Technology (Shanghai, China) produced the pAdeno-Tnnt2-mAG-hGeminin (1/110) adenovirus and determined the titer of adenovirus in HEK293 cells. They used the AdMax packaging system to produce adenovirus, which the Cre/loxP (or FLP/frt) recombinase was used to recombine shuttle plasmid carrying foreign genes and backbone plasmid in HEK293 cells. After cells appeared in cytopathic effect state, we collected cell supernatants and cell lysates, and purified the virus by cesium chloride density gradient centrifugation.

19. One day before transfection, seed HEK293 cells into 6-well plates and control the density of cells at 70%–80% when transfected.
20. Take out the cell culture plate 1 h before transfection, remove the cell culture medium, add 1.5 mL Opti MEM medium, and return plates into the incubator.
21. Prepare DNA-transfection reagent complex.
  a. Dissolve 4 μg viral vector plasmid (backbone plasmid: shuttle plasmid = 1:1) in Opti-MEM medium with a total volume of 250 μL and mixed gently.
  b. Dissolve the transfection reagent in Opti-MEM medium with a total volume of 250 μL and mix gently.
22. Add the transfection reagent diluents to the plasmid diluent, mix it gently while adding, and then place it at room temperature (18°C–26°C) for 20 min.
23. Take out the cell culture plates, add the DNA transfection reagent complex prepared above to the cell culture plates and put it back into the incubator.
24. After 6 h, aspirate the culture medium, wash once with PBS, and add 2 mL of fresh complete medium.
25. Change the culture medium every three days. Viral plaques might appear in about 7–15 days, and collect the supernatants after complete lesions.

### Virus amplification and purification

**Timing: 1 week**

26. Seed HEK293 cells on 30–40 10-cm dishes, and when the cells grow to 70%–80%, add 10 μL virus (about 10^7^–10^8^ PFU/mL) to each plate to infect the cells.
27. After 2–3 days.
  a. Add 500 μL 10% Nonidet P40 (NP40) to each plate to lyse cells.
  b. Collect cell lysates, centrifuge at 7,000 g for 10 min, discard cell fragments and collect supernatants.
28. Add 50 mL virus precipitation solution (20% PEG8000, 2.5 M NaCl) per 100 mL supernatants, and place on ice for 1 h to precipitate the virus.
29. Centrifuge the above mixture at 7,000 g for 20 min, discard the supernatant.
  a. Suspend the pellets in 10 mL CsCl solution with a density of 1.10 g/mL (solvent is 20 mM Tris-HCl, pH 8.0).
  b. Add 2.0 mL of 1.40 g/mL CsCl solution to a Beckman ultracentrifuge tube.
  c. Add another 3.0 mL of 1.30 g/mL CsCl solution.
30. Add 5 mL of virus suspension.
31. Centrifuge at 159,800 g for 2.5 h at 4°C.
32. Collect Viral bands with a density between 1.30–1.40 g/mL into a dialysis bag (boil the bag with 10 mM EDTA-Na2 for 10 min before use).
33. Dialysis buffer was dialyzed overnight (12–18 h) at 4°C, while changing the dialysate once in the middle.
34. Collect and store the virus at −80°C.

### Obtain the MADM reporter plasmid

**Timing: 5 weeks**

The MADM system consists of two knock-in genes, which are chimeric genes consisting of the N- and C-terminal of two different fluorescent proteins, such as RFP and GFP. The chimeric N-RFP/C-GFP or N-GFP/C-RFP alleles produce two non-functional proteins before Cre-mediated recombination. During the S phase of mitosis, sister chromatids replicate and pair. If inter-chromosomal recombination occurs, functional GFP and/or RFP are produced. Green and red daughter cells or yellow and colorless daughter cells are generated after mitosis^5^^,^^6^ (Figure 1B). This reporter further evaluates whether small molecules promote cardiomyocyte cytokinesis by infecting cardiomyocyte-specific Tnnt2-cre adenovirus in MADM^TG/GT^ mice.

35. Preparation of H202 vector is the same as step 1.
36. Amplify the DNA plasmids containing the Tnnt2 promoter and Cre cDNA sequences that are available in the laboratory (Figure 4A) (seen in Data S1).

**Figure 4:**
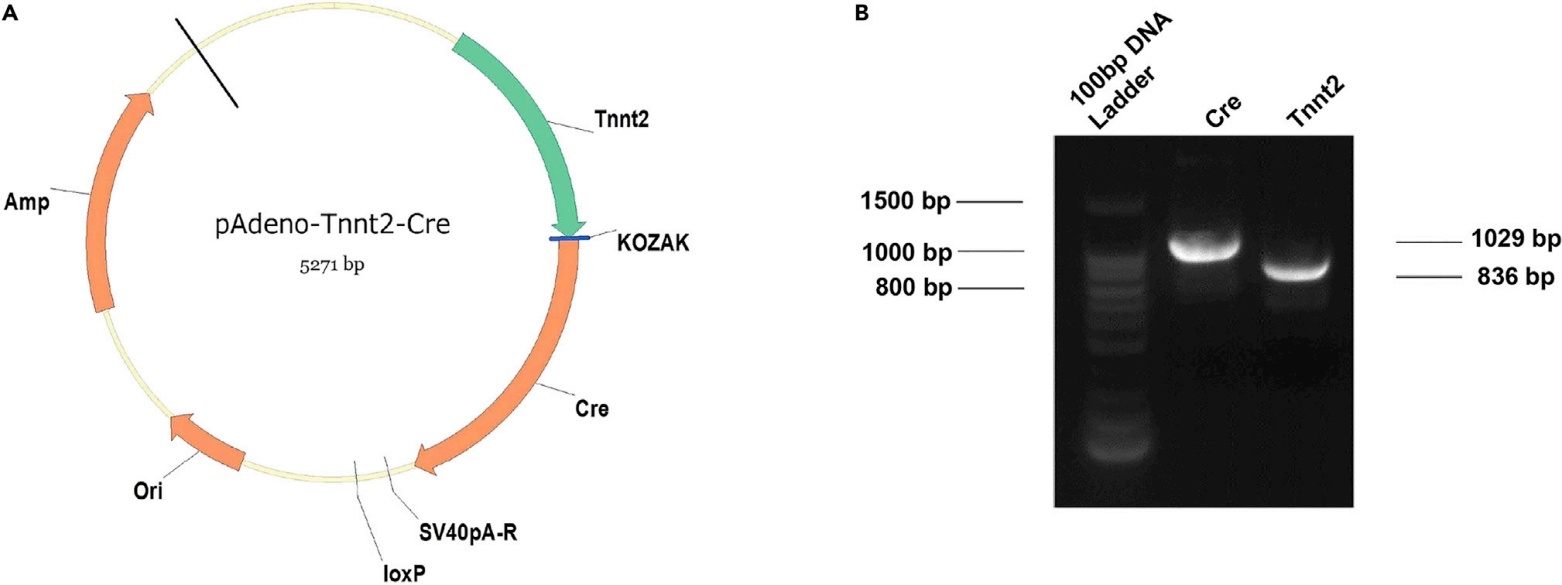
Construction of pAdeno-Tnnt2-Cre plasmid (A) Map of pAdeno-Tnnt2-Cre plasmid. Cre is driven by the Tnnt2 promoter and cloned into H202 vector. (B) Agarose gel electrophoresis resolving the Tnnt2 promoter and Cre fragments. Lane 1, 100bp DNA size marker; Lane 2, Cre fragment: the Cre fragment weight is in the 1029 bp; lane 3, Tnnt2 fragment, the Tnnt2 fragment weight is in the 836bp.
37. Extract and identify the Tnnt2 and Cre fragments by agarose gel electrophoresis (Figure 4B).
38. Design PCR primers with overlapping sequences for the Tnnt2 promoter, Cre cDNA, and H202 vector (Table 2), the length of overlapping sequences is 20 bp.
39. Carry out PCR reactions using the primers (see Table 2 for primers, Tables 3 and 4 for the PCR system and program settings).
40. Extract and identify the DNA fragments by agarose gel electrophoresis.
41. Clone Tnnt2 and Cre fragments into the H202 vector using the NEBuilder HiFi DNA assembly reaction system (Table 5).
42. Plasmid transformation is the same as steps 9–18.
43. Extract and identify the plasmids by agarose gel electrophoresis, and confirm the Tnnt2-Cre plasmid by Sanger sequencing (Table 2).
44. OBiO Technology (Shanghai, China) produced the Tnnt2-Cre adenovirus. The packaging, identification, amplification and purification procedures of pAdeno-Tnnt2-cre virus is the same as steps 19–34.

### Preparation of MADM-ML-11^TG/GT^ mice

**Timing: 8 weeks**

***Note:*** Dr. Chong Liu at the College of Medicine, Zhejiang University (Hangzhou, China) kindly provided the MADM-ML-11^TG^ and MADM-ML-11^GT^ mouse lines.^2^

45. Genotype the homozygous MADM-ML-11^TG^ and MADM-ML-11^GT^ mice by PCR (Table 2).
46. Generate MADM-ML-11^TG/GT^ mice by crossing homozygous MADM-ML-11^TG^ mice with homozygous MADM-ML-11^GT^ mice.

### Assessing the efficacy and specificity of Tnnt2-cre-mediated labeling of cardiomyocytes in MADM^TG/GT^ mice

**Timing: 1 week**

47. Isolate cardiomyocytes from P3 MADM^TG/GT^ mice.
48. Infect mouse cardiomyocytes with Tnnt2-cre adenovirus at an MOI of 100, 200, 500, or 1,000.
49. Identify the optimal infection titer of Tnnt2-cre adenovirus when cycling cardiomyocytes are labeled with RFP or GFP while quiescent cardiomyocytes are labeled with both RFP and GFP.
50. Infect P3 MADM^TG/GT^ mouse cardiomyocytes and fibroblasts with Tnnt2-cre adenovirus; The Tnnt2-cre-mediated labeling is specific to cardiomyocytes, but not fibroblasts, as shown in Figure 5.

**Figure 5:**
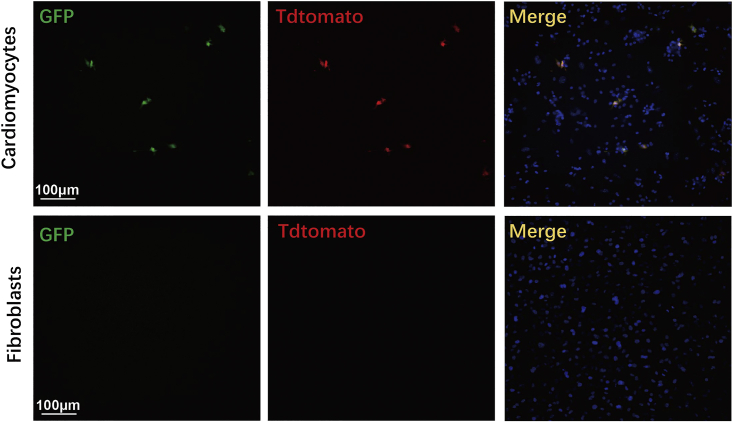
Fluorescence signals are evident in cardiomyocytes but not in fibroblasts at 96 h after adenovirus infection, noting the specific expression of GFP and Tdtomato fluorescent proteins only in cardiomyocytes Scale bars, 100 μm.
51. Document fluorescence at 24, 48, 72, 96, 120, and 144 h after adenovirus infection to determine the optimal time point for imaging and analysis. Fluorescence expression can be detected 96 h after infection (Figure 6).

**Figure 6:**
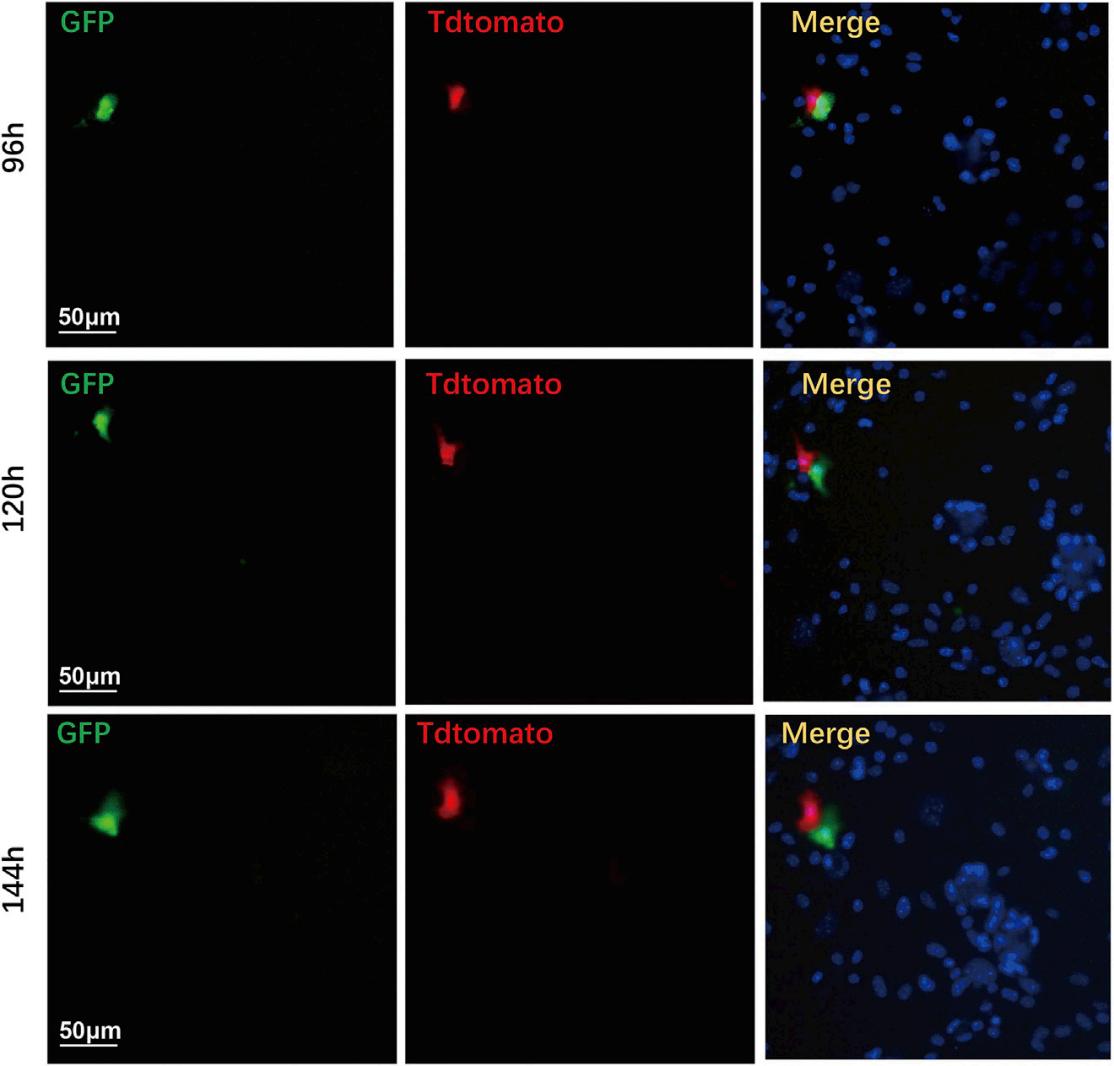
Dividing cardiomyocytes are shown from 96 to 144 h after adenovirus infection Compared with 96 h, the distance between GFP-positive and Tdtomato-positive cardiomyocytes increased at 120 h and 144 h, indicating that dividing cardiomyocytes are slowly separating. Scale bars, 50 μm.

## Key resources table

**Table:** 

REAGENT or RESOURCE	SOURCE	IDENTIFIER
**Bacterial and viral strains**		

pAdeno-Tnnt2-Cre	OBiO Technology (Shanghai, China)	N/A
pAdeno-Tnnt2-mAG-hGeminin (1/110)	OBiO Technology (Shanghai, China)	N/A
H202 pAdeno-MCMV-3Flag-IRES2-EGFP	OBiO Technology (Shanghai, China)	N/A

**Chemicals, peptides, and recombinant proteins**		

∼3,156 compounds	MCE	Cat#HY-L035
∼8,000 compounds	Cambridge	N/A
NaCl	Harveybio	Cat#SR1742
Yeast extraction	Oxoid	Cat#LP0021
Tryptone	Oxoid	Cat#LP0042
NaOH	Saitong	Cat#S20006
Agar power	SCR	Cat#10000561
Agarose	Biowest	Cat#111860
Sucrose	Titan	Cat#01090335
MgCl_2_·6H_2_O	SCR	Cat#10012818
PEG8000	Sangon	Cat#600433
Nonidet P40	Sangon	Cat#600385-0100
CsCl	Sigma	Cat#900481-100
EDTA-2Na	SCR	Cat#10009717
Tris	Sangon	Cat#A610195-0500
HCl	SCR	Cat#10011018
Ampicillin sodium salt	VWR	Cat#0339-25G
Cytosine arabinoside	Vetec	Cat#V900339
Collagenase II	Gibco	Cat#17101015
Trypsin	Amresco	Cat#VWRV0785
0.25% Trypsin-EDTA (1×)	Gibco	Cat#25200-056
XbaI restriction enzymes	NEB	Cat#R0145
AgeI-HF restriction enzymes	NEB	Cat#R3552
PrimeSTAR Max DNA Polymerase	Takara	Cat#R045A
BSA	MP Biomedicals	Cat#180728
Horse serum	Gibco	Cat#16050122
Fetal bovine serum (FBS)	Yeasen	Cat#40130ES76

**Critical commercial assays**		

EndoFree Maxi Plasmid Kit	Tiangen	Cat#DP117
Universal DNA purification kit	Tiangen	Cat#DP204
Cutsmart buffer	NEB	Cat#B6004
NEBuilder HiFi DNA Assembly Master Mix	NEB	Cat#E2621
10×PBS	Solarbio	Cat#P1022
Hoechst 33342	Yeasen	Cat#40732ES03
100 bp DNA ladder	Genstar	Cat#M116
100×Penicillin-streptomycin	Solarbio	Cat#P1400
DMSO	Sigma-Aldrich	Cat#D8418
0.4% trypan blue	Solarbio	Cat#C0040
HBSS without Ca^2+^ and Mg^2+^	MacGene	Cat#CC016
DMEM high glucose medium	Hyclone	Cat#SH30022.01
Opti-MEM I	Gibco	Cat#31985070

**Experimental models: Cell lines**		

HEK293	OBiO Technology (Shanghai, China)	N/A

**Experimental models: Organisms/strains**		

Sprague-Dawley rat: wild-type, postnatal day 3 (P3), both male and female	Vital River Laboratory Animal Technology Co., Ltd (Beijing, China)	N/A
Mouse: MADM-ML-11^TG^, sexually mature, both male and female	Liu et al.^2^	N/A
Mouse: MADM-ML-11^GT^, sexually mature, both male and female	Liu et al.^2^	N/A
Mouse: MADM-ML-11^TG/GT^, postnatal day 3 (P3), both male and female	Liu et al.^2^	N/A

**Oligonucleotides**		

Primers for pAdeno-Tnnt2-mAG-hGeminin (1/110)	This paper	See Table 2
Primers for pAdeno-Tnnt2-Cre	This paper	See Table 2
Primers for MADM-ML-11^TG^ and MADM-ML-11^GT^ mice genotyping	The Jackson Laboratory	See Table 2

**Recombinant DNA**		

Plasmid: Tnnt2-Cre	This paper	N/A
Plasmid: MCMV-MCS-3×FLAG-IRES2-EGFP	This paper	N/A
Plasmid: Tnnt2-mAG-hGeminin (1/110)	This paper	N/A

**Software and algorithms**		

GraphPad Prism 8.3.0	GraphPad	https://www.graphpad.com/scientific-software/prism/
MetaXpress software	Molecular Devices	https://www.moleculardevices.com/products/cellular-imaging-systems/acquisition-and-analysis-software/metaxpress
Harmony	PerkinElmer	N/A

**Other**		

ECHO 520	Labcyte	Cat#001-10080
Multidrop^TM^ Combi	Thermo Scientific	Cat#5840320
Opera Phenix	PerkinElmer	N/A
ImageXpress Micro XL Imaging System	Molecular Devices	N/A
CO_2_ incubator	Thermo Scientific	Cat#3111
Magnetic heating stirrer	IKA	Cat#0003810001
96-well plates	Corning	Cat#3599
384-well polypropylene microplate	Labcyte	Cat#P05525
Plate sealing films	Axygen	Cat#HS-400
Countess II Automated Cell Counter	Invitrogen	Cat#AMQAX1000
Inverted fluorescence microscope	Olympus	Cat#IX71
NanoDrop 2000 Ultra Micro Spectrophotometer	Thermo Scientific	N/A
Milli Q Plus Ultrapure Water System	Millipore	N/A
Ultracentrifuge	Hitachi	Cat#CP70ME
Centrifuge	Eppendorf	Cat#5810
15 mL centrifuge tubes	Biofil	Cat#CFT011150
50 mL centrifuge tubes	Biofil	Cat#CFT011500
Ultracentrifuge tube	Hitachi	Cat#332901A
Dialysis bag	Yuanye	Cat#MD3544-5M
Straight forceps	Harveybio	Cat#MB1663
Curved forceps	Harveybio	Cat#MB1664
Straight ophthalmic scissors	Jinzhong	Cat#Y00030
Curved ophthalmic scissors	Jinzhong	Cat#Y00040
100 mm plastic dishes	Corning	Cat#430167
Electronic pipettes	Rainin	Cat#17005916
100 μm cell strainers	Biologix	Cat#15-1100
0.22 μm filter	Millipore	Cat#SLGPR33RB
10 mL syringes	TNTC	Cat#1870
20 mL bottle	Wheaton	Cat#225288
1.5 cm rotor	TNTC	Cat#2363
Gauzes	Solarbio	Cat#YA0720

***Alternatives:*** forceps, ophthalmic scissors, bottle, rotor and gauzes are alternative. Sterilize these materials by high pressure steam before use. Besides, using other brands of syringes, pipettes, sterilized centrifuge tubes and cell counter is permissible.

## Materials and equipment

**Table:** LB medium

Reagent	Final concentration	Amount
Tryptone	10 g/L	10 g
Yeast extract	5 g/L	5 g
NaCl	10 g/L	10 g
ddH_2_O	N/A	1 L
NaOH	N/A	to pH=7.4
**Total**	**N/A**	**1 L**

Store tryptone, yeast extract, NaCl and NaOH power at room temperature (18°C–26°C) for up to 2 years. Sterilize the LB medium after preparation. Add ampicillin to make the final concentration at 100 μg/mL before use. Store sterilized LB medium at 4°C for up to 3 weeks.

**Table:** LB solid medium

Reagent	Final concentration	Amount
Tryptone	10 g/L	10 g
Yeast extract	5 g/L	5 g
NaCl	10 g/L	10 g
Agar	15 g/L	15 g
ddH_2_O	N/A	1 L
NaOH	N/A	to pH=7.4
**Total**	**N/A**	**1 L**

Store Agar power at room temperature (18°C–26°C) for up to 2 years. Sterilize LB medium by pressured steam. After sterilization, add ampicillin to make the final concentration at 100 μg/mL when the medium is 50°C–60°C. Mix and pour into 10 cm culture dish to wait for solidification. Store LB plate at 4°C for up to 3 weeks.

**Table:** 1 M Tris-HCl (pH=8.0)

Reagent	Final concentration	Amount
Tris	1 mol/L	121.1 g
ddH2O	N/A	950 mL
HCl	N/A	to pH=8.0 (25°C)
**Total**	**N/A**	**1 L**

Store tris and HCl at room temperature (18°C–26°C) for up to 3 years. Sterilize 1 M Tris-HCl solution and store at room temperature for up to 1 year.

**Table:** Dialysis buffer

Reagent	Final concentration	Amount
Sucrose	50 g/L	50 g
1 M Tris-HCl	0.01 mol/L	10 mL
MgCl_2_·6H_2_O	0.002 mol/L	0.4066 g
ddH_2_O	N/A	1,000 mL
**Total**	**N/A**	**1 L**

Store sucrose, 1 M Tris-HCl and MgCl_2_·6H_2_O at room temperature (18°C–26°C) for up to 2 years. Prepare dialysis buffer at room temperature before use.

**Table:** Neonatal rat CM isolation digestion solution

Reagent	Final concentration	Amount
Collagenase II	0.3 mg/mL	0.0338 g
Trypsin	1 mg/mL	0.0638 g
HBSS	N/A	60 mL
**Total**	**N/A**	**60 mL**

Store collagenase II and trypsin powder at 4°C for up to 2 years. Store HBSS at room temperature (18°C–26°C) for up to 1 year. Prepared the neonatal rat CM isolation digestion solution freshly and put on ice before starting experiments.

**Table:** Neonatal mouse CM isolation digestion solution

Reagent	Final concentration	Amount
Collagenase II	0.5 mg/mL	0.015 g
BSA	5 mg/mL	0.15 g
Complete medium	N/A	30 mL
**Total**	**N/A**	**30 mL**

Store collagenase II and BSA powder at 4°C for up to 1 year. Prepare the neonatal mouse CM isolation digestion solution freshly and put on ice before starting experiments.

**Table:** Complete medium

Reagent	Final concentration	Amount
High glucose DMEM	89%	89 mL
FBS	10%	10 mL
Penicillin-streptomycin	1%	1 mL
**Total**	**100%**	**100 mL**

Store high glucose DMEM medium at 4°C for up to 1 year. Store FBS and Penicillin-streptomycin at −20°C for up to 1 year. Store complete medium at 4°C for up to 3 weeks.

**Table:** Culture medium

Reagent	Final concentration	Amount
High glucose DMEM	93.9%	93.9 mL
Horse serum	5%	5 mL
Penicillin-streptomycin	1%	1 mL
Cytosine arabinoside (20 mM)	0.1%	100 μL
**Total**	**100%**	**100 mL**

Store cytosine arabinoside (powder) at 4°C for up to 2 years, and cytosine arabinoside (solution) at –20°C for up to 1 month. Store horse serum at –20°C for up to 1 year. Store culture medium at 4°C for up to 3 weeks.

**Table:** Medium for adenovirus infection

Reagent	Final concentration	Amount
High glucose DMEM	98%	98 mL
FBS	1%	1 mL
Penicillin-streptomycin	1%	1 mL
**Total**	**100%**	**100 mL**

Store infection medium at 4°C for up to 3 weeks

**Table:** Medium for diluting small molecules

Reagent	Final concentration	Amount
High glucose DMEM	98.5%	98.5 mL
FBS	0.5%	500 μL
Penicillin-streptomycin	1%	1 mL
**Total**	**100%**	**100 mL**

Store diluting medium at 4°C for up to 3 weeks.

## Step-by-step method details

### Harvesting heart ventricles from neonatal rats

**Timing: 30 min**

This section describes the procedures for harvesting heart ventricles from neonatal rats, washing in pre-cooled PBS to remove the blood, atria, and other tissues.^3^ Perform all the following steps are in a sterile cell-culture hood, all surgical instruments should be sterile. Keep prepared reagents on ice and harvest the ventricles as fast as possible.

1. Prepare the following surgical instruments, consumables, and reagents.
  a. Surgical instruments.
    i. Two sterile straight forceps.
    ii. Two sterile curved forceps.
    iii. Two sterile straight scissors.
    iv. One sterile curved scissor.
  b. Consumables.
    i. 15 mL sterile centrifuge tubes.
    ii. 50 mL centrifuge tubes.
    iii. Two 100 μm cell strainers.
    iv. One 0.22 μm filter.
    v. 10 mL syringes.
    vi. One sterilized bottle.
    vii. One sterilized rotor.
    viii. Two sterilized gauzes.
    ix. Two sterilized 100 mm plastic dishes.
    x. 10-mL pipettes.
    xi. One carcass bag.
    xii. One ice box.
  c. Reagents.
    i. 75% alcohol.
    ii. Pre-cooled HBSS without Ca^2+^ and Mg^2+^ (MacGene, CC016).
2. Prepare 60 mL digestion solution containing collagenase II (0.3 mg/mL; Gibco, 2163320) and trypsin (1 mg/mL; Amresco, VWRV0785) in HBSS.

***Note:*** Filter digestion solution with a 0.22 μm filter using 10 mL syringes and place on ice.

**Figure 7:**
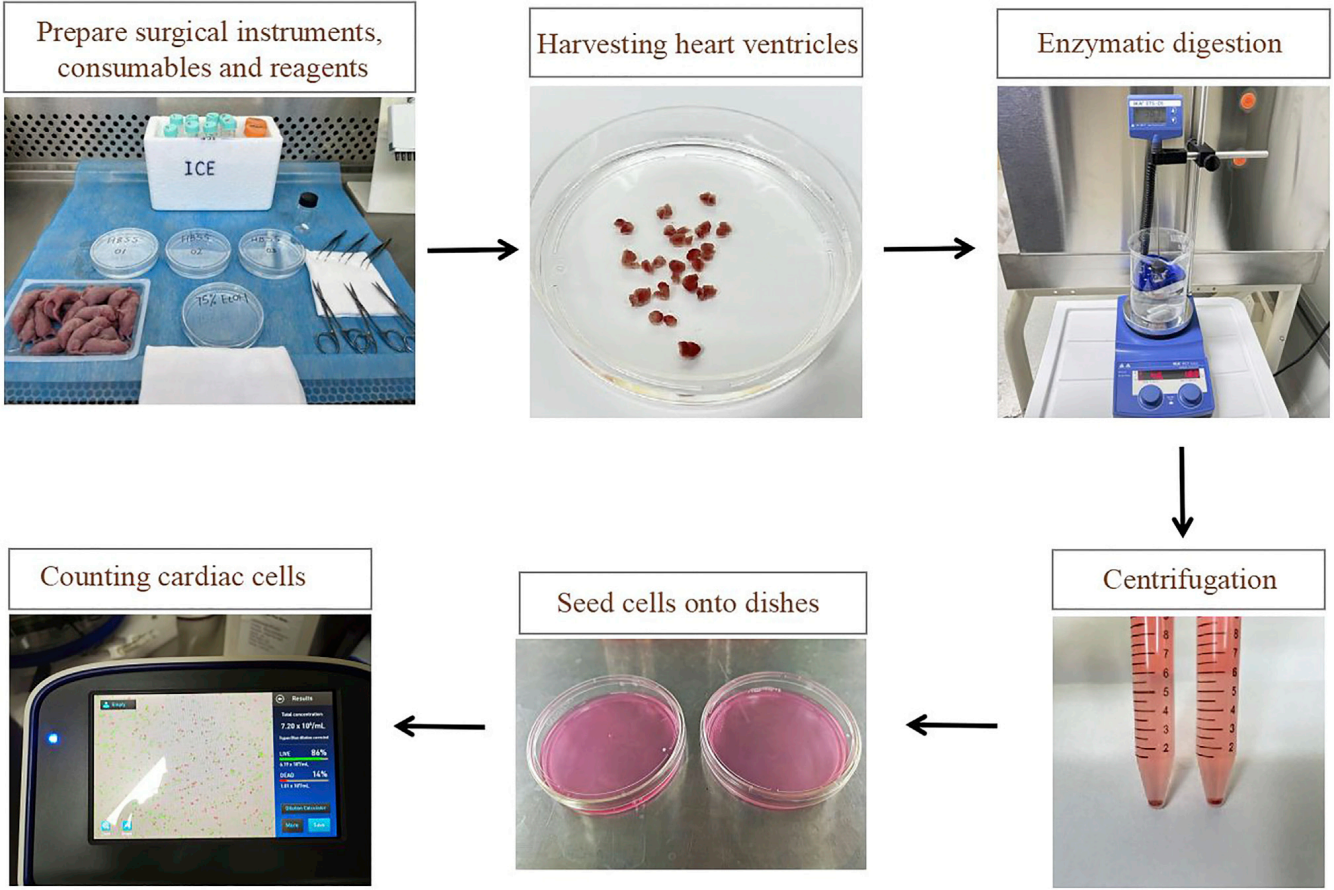
Schematic of neonatal rat cardiomyocyte isolation
4. Turn on the constant temperature magnetic stirrer, set the stir speed to 180 rpm, and the temperature to 37°C.

***Note:*** The constant temperature magnetic stirrer heats up slowly. In order to ensure that the heart can be digested at 37°C after harvesting, it needs to be turned on at least half an hour in advance.

3. Prepare 100 mL of high glucose DMEM (Hyclone, SH30022.01) containing 10% fetal bovine serum (FBS, Yeasen, 40130ES76) and 1% penicillin-streptomycin (Figure 7).

5. Transfer 15 mL pre-cooled HBSS without Ca^2+^ and Mg^2+^ into three sterile 100 mm dishes and place them on ice.
6. Place 3-day-old (P3) SD rats and 75% alcohol in two boxes, and cadavers in the third box.
7. Sterilize P3 SD rats with 75% alcohol, mainly focusing on the chest.
8. Decapitate pups using sterile straight scissors, and open the chest along the sternum to remove the heart.

***Note:*** A rat heart can obtain 4 × 10^6^ cardiomyocytes. The number of hearts can be calculated according to the total number of cardiomyocytes required.

9. Collect hearts with curved scissors.
  a. Immediately transfer into the 100 mm dish containing pre-cooled HBSS without Ca^2+^ and Mg^2+^.
  b. Transfer hearts to other two dishes containing HBSS with curved tweezers to remove the blood, atria, and other tissues (Figure 7).

### Enzymatic digestion of ventricles

**Timing: 1 h**

This section describes the procedures for enzymatic digestion of ventricles. Digest ventricles for 8–10 times and collect the supernatants of each digestion. Centrifuge the collected cells and seed the cell suspension onto two dishes.

10. Add 5 mL filtered digestion solution and a sterile rotor to the 10 mL sterilized bottle.
  a. Transfer the collected hearts into the bottle.
  b. Cut the hearts into small pieces using straight scissors.
  c. Digest the ventricles under 180 rpm agitation at 37°C for 5 min (Figure 7).

***Note:*** Do not cut the heart into too small pieces while avoiding the adherence of tissues during digestion.

11. After 5 min, leave the ventricle tissues to stand for a minute, discard the supernatant, and add a fresh 5-mL digestion solution.
12. Repeat steps 10 and 11 to remove the red cells.

***Note:*** Before the second digestion, cut the heart tissue into smaller pieces with straight scissors to fully remove the blood.

13. After the third digestion, transfer the supernatants to a 15 mL tube containing 5 mL high-glucose DMEM with 10% FBS and 1% penicillin-streptomycin.
  a. Pipette up and down 10 times using a 10 mL pipette to stop the digestion.
  b. Place the 15 mL tube on ice.

**CRITICAL:** Fully mix the supernatants and complete medium by pipetting up and down to terminate digestion.

14. Add 5 mL digestion solution to the remaining cardiac tissue and digest it under 180 rpm agitation at 37°C for 5 min.
15. Repeat steps 13 and 14 8–10 times with the remaining cardiac tissue until it is flocculent. Troubleshooting 1.
16. After finishing the last digestion, centrifuge cells of all 15 mL tubes at 200 g for 5 min (Figure 7).
17. Carefully discard the supernatant and re-suspend the remaining cell pellets in 20 mL high-glucose DMEM containing 10% FBS and 1% penicillin-streptomycin.
18. Pipette up and down using a 10 mL pipette to ensure a homogeneous solution of single cells.
19. Pass the cell suspension through a 100 μm cell strainer.
20. Seed onto two sterilized 100 mm plastic dishes to allow the fibroblasts to adhere.
21. Incubate the cells for 2 h at 37°C in a 5% CO_2_ humidified incubator.

***Note:*** This step is designed to separate non-cardiomyocytes from cardiomyocytes. Non-cardiomyocytes in the heart are mainly fibroblasts, and fibroblasts adhere to the culture plates more rapidly than cardiomyocytes. Using differential attachment technique, we allow the cells to adhere for two hours and then collect non-adhered cells for enriching cardiomyocytes.

### Counting and plating the cells

**Timing: 1 h**

These procedures are performed after culturing cardiac cells for 2 h.

22. After culture for 2 h.
  a. Gently wash the dishes with 10 mL high-glucose DMEM containing 10% FBS and 1% penicillin-streptomycin.
  b. Transfer the supernatant to two 15 mL tubes.
23. Centrifuge at 200 g for 5 min at 25°C.
24. Re-suspend the pellets in 10 mL high-glucose DMEM containing 10% FBS and 1% penicillin-streptomycin.
25. Pass the cell suspensions through a 100 μm cell strainer.
26. Take 9 μL cell suspension and 1 μL 0.4% trypan blue (Solarbio, C0040), pipette up and down to mix them, and stain for 3 min.
27. Load 10 μL of the mixture into the well of a cell-counting chamber.
28. Determine the total cell number and viability by a Countess II Automated Cell Counter.
29. Prepare culture medium containing high glucose DMEM, 20 μM cytosine arabinoside, and 5% horse serum.

***Note:*** Since cytosine arabinoside blocks DNA synthesis to inhibit non-cardiomyocytes proliferation, thus avoiding the dominant growth of non-cardiomyocytes during adherent culture.

30. Add a certain amount of culture medium according to cell viability and total cell number.
  a. Pipette up and down using a 10 mL pipette to ensure a homogeneous solution of single cells.
  b. Seed the cell suspensions on 96-well plates at 15,000 cells per well and incubate at 37°C and 5% CO_2_.

### Adenoviral infection

**Timing: 30 min**

This section describes the procedures for pAdeno-Tnnt2-mAG-hGeminin (1/110) adenovirus infection in neonatal rat ventricular myocytes (NRVMs).

**Figure 8:**
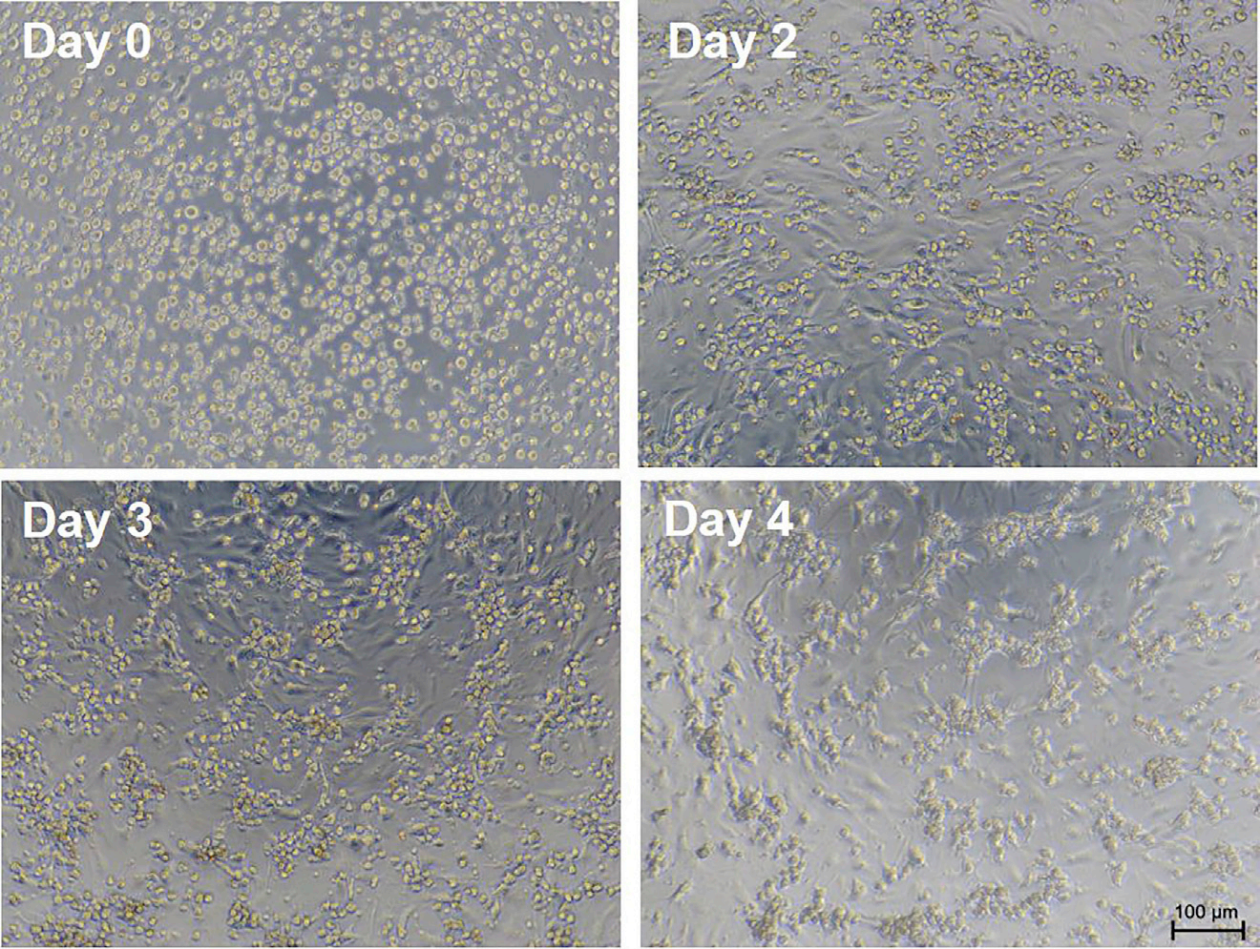
Bright-field images of cardiomyocytes at different culture times Scale bar, 100 μm.
32. Calculate the required amount of virus according to the following formula. The amount of virus for each well (μL) = (MOI) × (the number of cells at the time of infection) / the titer (PFU/mL) × 1000.
33. Remove the medium from the 96-well plate.
34. Add pAdeno-Tnnt2-mAG-hGeminin (1/110) adenovirus at an MOI of 100 that are diluted in a high-glucose medium containing 1% FBS. Troubleshooting 3 and 4.

***Note:*** Adenovirus should be stored as aliquots to avoid repeated freezing and thawing. After taking out the virus, place the adenovirus on ice immediately. Mix adenovirus thoroughly by pipetting up and down before adding it to the medium.

31. After plating for 48 h, confirm that NRVMs are attached and exhibit spontaneous beating under microscopy (Figure 8). Troubleshooting 2.

### Small-molecule treatment

**Timing: 2 h**

This section describes the procedures of small-molecule treatment. All the following steps are performed in a sterile cell-culture hood, except for transferring compounds by ECHO 520.

35. Take 384-well chemical library plates from a –80°C refrigerator at least 2 h in advance.
  a. Thaw compounds at room temperature (18°C–26°C).
  b. Centrifuge them quickly with a centrifuge dedicated to these plates.
36. Prepare high-glucose DMEM with 1% penicillin-streptomycin and 0.5% FBS and as negative control (0.5% FBS with DMSO) or 10% FBS as positive control (10% FBS with DMSO).
37. After adenoviral infection for 24 h.
  a. Aspirate the culture medium.
  b. Immediately add fresh culture medium with 2 μM indicated small molecules.

**CRITICAL:** Do not discard the liquid in several 96-well plates at one time to avoid a great impact on the cell state caused by a long operating time. When transferring compounds with ECHO 520, the plates need to be inverted, so it is necessary to ensure that there is no liquid remaining in the plates.

38. Transfer compounds in 384-well plates in a volume of 20 nL (10 mM) to 96-well plates using ECHO 520 (Labcyte) (Figure 9).

**Figure 9:**
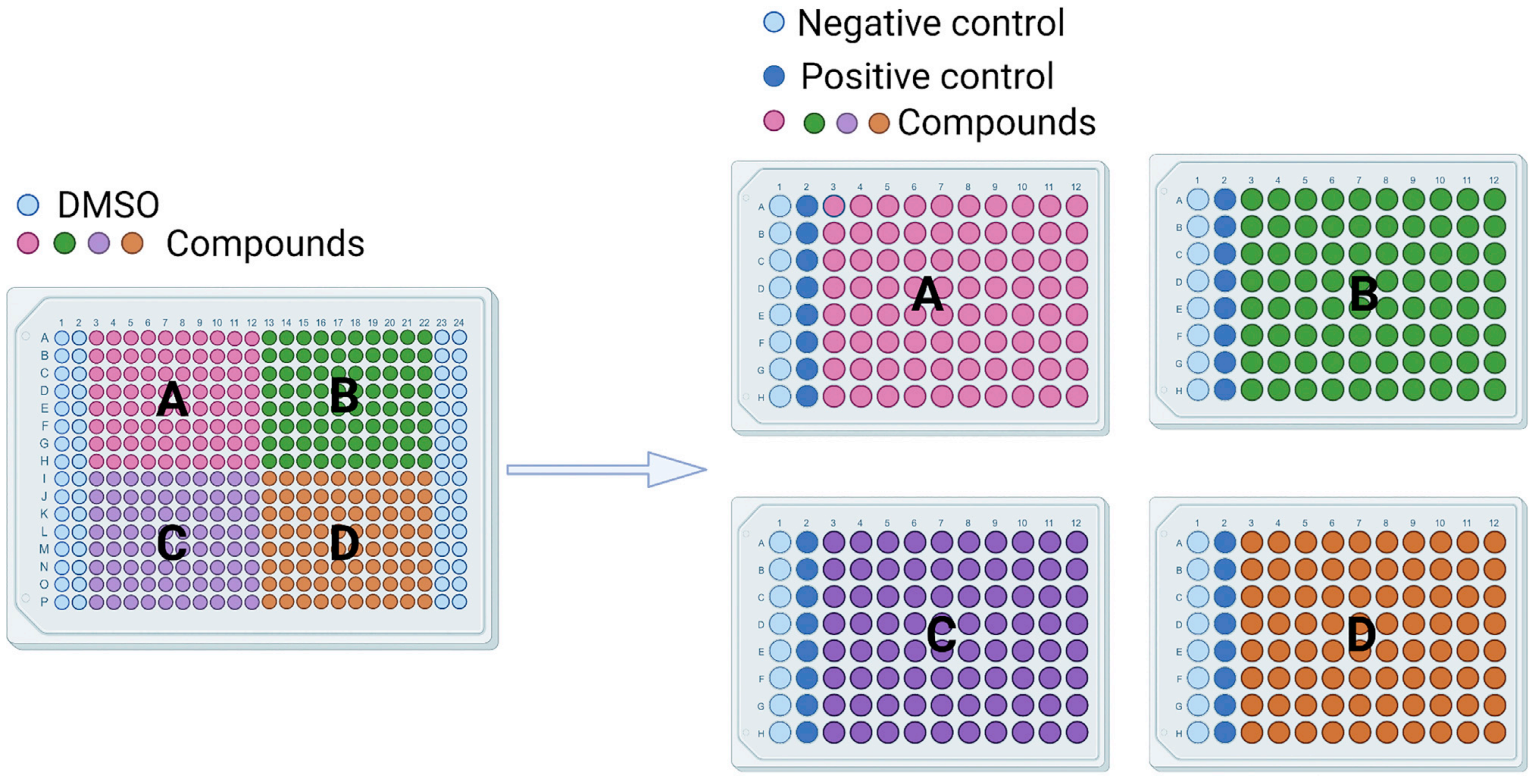
Schematic of the location of small molecules from one 384-well plate to four 96-well plates
39. Distribute 100 μL DMEM with 0.5% FBS and 1% penicillin-streptomycin to each well using Multidrop Combi (Thermo Scientific), thus making a final working concentration of 2 μM for each compound.
40. Add both negative control (0.5% FBS with DMSO) and positive control (10% FBS with DMSO) in the 96-well plates as shown in Figure 9.
41. Immediately place the 96-well plate into the incubator after adding compounds and negative and positive controls.
42. Repeat steps 37–41.
43. Seal the 384-well library plates with sealing film, flat with a roller, and return to the –80°C refrigerator.

### Imaging and analysis

**Timing: 2–4 h**

This section describes the procedures for imaging and analysis of FUCCI-positive NRVMs after small-molecule treatment for 24 h.

44. After small-molecule treatment for 24 h, aspirate the culture medium, and stain the nuclei with Hoechst 33342.
45. Capture images of FUCCI [mAG-hGeminin (1/110)]-positive green fluorescent NRVMs using the MD ImageXpress Micro XL Imaging System (Molecular Devices). (refer to Table 6 for parameter settings of MD ImageXpress Micro XL Imaging System). Troubleshooting 5.

**Table 6:** Parameter settings of MD ImageXpress Micro XL Imaging System

Parameter	Setting
Objective magnification	20×
Number of fields	9–25
Depth of focus	3.5 μm
Number of cells	> 3,000 cells per well >10,000 cells per well (for MADM system)
Resolution	1280 × 1080
Image size	842.64 × 710.98 μm
Binning	2
Channel 1	Excitation: 377 nm, Emission: 447 nm, Exposure: 150 ms
Channel 2	Excitation: 482 nm, Emission: 536 nm, Exposure: 150–200 ms
Channel 3 (for MADM system)	Excitation: 562 nm, Emission: 624 nm, Exposure: 150–200 ms

**Table 7:** Parameter settings of PE Opera Phenix

Parameter	Setting
Objective magnification	20×
Number of fields	9–25
Depth of focus	4.4 μm
Number of cells	> 3,000 cells per well >10,000 cells per well (for MADM system)
Resolution	1080 × 1080
Image size	650 × 650 μm
Binning	2
Channel 1	Excitation: 355–385 nm, Emission: 430–500 nm, Exposure: 10 ms
Channel 2	Excitation: 460–490 nm, Emission: 500–550 nm, Exposure: 75–200 ms
Channel 3 (for MADM system)	Excitation: 530–560 nm, Emission: 570–650 nm, Exposure: 100–300 ms

***Note:*** Acquiring at least 9 images per well and analyzing 3,000 cells per well.

***Optional:*** Images of FUCCI [mAG-hGeminin (1/110)]-positive green fluorescent NRVMs can also be acquired using Opera Phenix (Perkin Elmer) (refer to Table 7 for parameter settings of PE Opera Phenix) and the data can be analyzed using the harmony software in Opera Phenix.

46. Count the number of FUCCI-positive NRVMs and the total number of NRVMs based on cell segmentation using MetaXpress software.
47. Calculate the percentage of FUCCI-positive NRVMs in each well.
48. Determine positive hits of small molecules based on at least 2-fold greater effects on the induction of FUCCI-positive NRVMs than the negative control (Figure 10). Troubleshooting 6.

**Figure 10:**
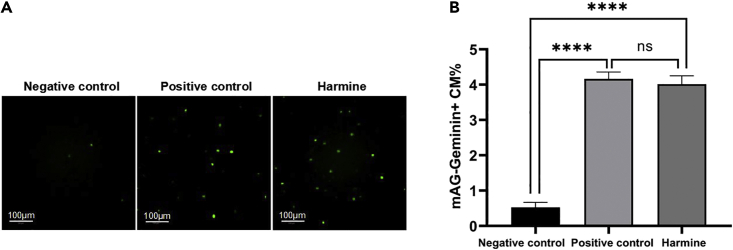
The compound Harmine enables neonatal rat cardiomyocyte proliferation (A) Representative images of P3 NRVMs infected with FUCCI [Ad-Tnnt2-mAG-hGeminin (1/110)] adenovirus and treated with either DMSO (negative control), 10% FBS (positive control), or Harmine. Scale bars, 100 μm. (B) Quantitative analysis of data from (A). n = 3,000 cells per replicate, 4 replicates per group. Data are the mean ± SEM. ∗∗∗∗p < 0.0001; ns, not significant; unpaired, two-tailed Student’s t test.
49. Confirm candidate small molecules (2 μM) twice to assess their effects on induction of FUCCI-positive NRVMs. Troubleshooting 7.

### Determine optimal concentration of candidate compounds

**Timing: 1–2 weeks**

The concentration of candidate compounds in the three repeated initial screenings are 2 μmol/L, the following steps describe procedures for determining the optimal working concentration for each candidate.

50. Isolate and seed P3 NRVMs on 24-well plates at 50,000 cells per well.
51. After 48 h, infect NRVMs with pAdeno-Tnnt2-mAG-hGeminin (1/110) adenovirus at an MOI of 100.
52. Dissolve each candidate small molecule in DMSO at 10 mM.
53. Dilute these small molecules into high-glucose DMEM containing 0.5% FBS at 0, 0.5, 1, 2, 5, or 10 μM and are applied to NRVMs for 24 h.

***Note:*** Vortex small molecules before and after dilution into high-glucose DMEM containing 0.5% FBS. Gently shake the 24-well plates after adding small molecules to cardiomyocytes.

54. Measure the percentage of FUCCI-positive NRVMs in each well based on cell segmentation using MetaXpress software. Troubleshooting 8 and 9.

### Harvesting neonatal mouse ventricles

**Timing: 12–16 h**

This section describes the procedures of isolating cardiomyocytes from neonatal mouse ventricles with overnight pre-digestion by trypsin.

***Note:*** All the following steps are performed in a sterile cell-culture hood, all surgical instruments should be sterile, and the prepared reagents should be kept on ice.

55. Prepare the following surgical instruments, consumables, and reagents.
  a. Surgical instruments.
    i. Three sterile curved forceps.
    ii. One straight sterile scissor.
    iii. One sterile curved scissor.
  b. Consumables.
    i. One 0.22 μm filter.
    ii. 10 mL syringes.
    iii. One sterilized bottle.
    iv. One sterilized rotor.
    v. Two sterilized gauzes.
    vi. Three sterilized 100 mm plastic dishes.
    vii. 10 mL pipettes.
    viii. One carcass bag.
    ix. One ice box.
  c. Reagents.
    i. 75% alcohol.
    ii. Pre-cooled HBSS without Ca^2+^ and Mg^2+^.
56. Add 10 mL filtered 0.25% trypsin with EDTA and a rotor to a sterilized bottle. Place the bottle on ice.
57. Transfer 15 mL pre-cooled HBSS without Ca^2+^ and Mg^2+^ into three sterile 100 mm dishes and place them on ice.
58. Place P3 MADM-ML-11^TG/GT^ neonatal mice and 75% alcohol in two boxes, and cadavers in the third box.
59. Sterilize P3 MADM-ML-11^TG/GT^ mice with 75% alcohol, mainly focusing on the chest.
  a. Following euthanasia, open the chest of pups along the sternum.
  b. Use the curved scissors to collect the ventricles.

***Note:*** A mouse heart can isolate 2 × 10^6^ cardiomyocytes. The number of hearts can be calculated according to the total number of cardiomyocytes required.

60. Transfer each heart immediately into the 100 mm dish containing pre-cooled HBSS without Ca^2+^ and Mg^2+^.
61. Using the curved tweezers, transfer hearts to the second and then third dish to remove the blood and other tissues.
62. Using straight scissors to make an incision in the heart, which does not need to be completely cut apart.
63. Gently transfer hearts to the bottle containing 0.25% trypsin with EDTA.
64. Place the bottle in a 4°C refrigerator and leave overnight (12–16 h).

### Digesting, counting, and plating mouse CMs

**Timing: 4 h**

This section describes the procedures of isolating mouse cardiomyocytes. Ventricles are digested by collagenase II 3–4 times. To separate the cardiomyocytes from non-cardiomyocytes, the cells are plated into 2 dishes and incubated for 2 h. After that, purified cardiomyocytes are seeded into culture plates.^7^

65. After pre-digestion, prepare the following surgical instruments, consumables.
  a. Surgical instruments.
    i. Sterile straight scissors.
  b. Consumables.
    i. Two 100 μm cell strainers.
    ii. 15 mL sterile centrifuge tubes.
    iii. 50 mL centrifuge tubes.
    iv. One 0.22 μm filter.
    v. 10 mL syringes.
    vi. One ice box.
66. Prepare 60 mL complete medium of high glucose DMEM containing 10% FBS and 1% penicillin-streptomycin.
67. Prepare 30 mL digestion solution containing collagenase II (0.5 mg/mL; Gibco, 2163320) and BSA (5 mg/mL; MP Biomedicals, Y180306) in complete medium.
68. Filter the digestion solution with a 0.22 μm filter and place on ice.
69. Turn on the constant temperature magnetic stirrer, set the stir speed to 90 rpm, and the temperature to 37°C.
70. Take the bottle out of the 4°C refrigerator.
  a. Add 10 mL complete medium to the bottle.
  b. Put the bottle in a water bath under 90 rpm agitation at 37°C for 5 min to stop the trypsin digestion.
71. After 5 min, discard the supernatant and add 5 mL fresh digestion solution.
72. Digest the heart in the water bath under 90 rpm agitation at 37°C for 1 min.
73. After 1 min, discard the supernatant and add fresh 5 mL digestion solution.
74. Digest the heart in the water bath under 90 rpm agitation at 37°C for 5 min.
75. After 5 min, gently transfer the supernatants into a 15 mL centrifuge tube and place the tube on ice, being careful not to transfer tissue into the tube.
76. Repeat steps 73 and 75 3–4 times until the tissue is flocculent.
77. After finishing digestion, centrifuge all cells in the 15 mL tubes at 200 g for 5 min.
78. Carefully discard the supernatants and re-suspend the cell pellets in 20 mL complete medium by pipetting up and down.
79. Pass the cell suspensions through a 100 μm strainer.
80. Seed cell suspensions onto two sterilized 100 mm plastic dishes.
81. Incubate cells for 2 h at 37°C in a 5% CO_2_ humidified incubator to separate cardiomyocytes from non-cardiomyocytes.
82. After 2 h incubation.
  a. Harvest the cell suspension and gently wash the dishes with 10 mL complete medium.
  b. Transfer the suspensions into two 15 mL tubes.
83. Centrifuge at 200 g for 5 min at room temperature (18°C–26°C).
84. Gently re-suspend the cell pellets in complete medium by pipetting.
85. Pass the cell suspension through a 100 μm strainer.
86. Count the cells as in steps 26 and 28.
87. Prepare high glucose DMEM containing 20 μM cytosine arabinoside, 10% FBS, and 1% penicillin-streptomycin.
88. Seed the cardiomyocyte on 96-well plates at 15,000 cells per well and incubate at 37°C, 5% CO_2_.

### Tnnt2-Cre adenoviral infection and candidate small molecule treatment

**Timing: 30 min for infection and 2 h for compound treatment**

This section describes the procedures of Tnnt2-Cre adenoviral infection and the procedures of small-molecule treatment, which induces inter-chromosomal recombination in MADM cardiomyocytes and collect high-content microscopy images to evaluate the efficiency of cytokinesis.

89. After 48 h in culture.
  a. Prepare the high glucose DMEM containing 1% FBS and 1% penicillin streptomycin.
  b. Dilute pAdeno-Tnnt2-Cre adenovirus at an MOI of 200 in the medium. Follow infection protocol with steps 31–34.
90. After 24 h of infection, prepare preheated adequate DMEM containing 0.5% FBS and 1% penicillin-streptomycin.
91. Dilute candidate compounds at the optimal concentration in the medium.
92. Add equivalent DMSO to the medium (0.5% FBS) as a negative control.
93. Gently remove the medium from the 96-well plate by pipetting, and add negative control and candidate compound media to the plates.
94. After incubation for 72 h, stain the nuclei with Hoechst 33342.
95. Capture images and analyze the data using MD ImageXpress Micro XL Imaging System (Figure 11). Imaging parameter settings could refer to step 45.

**Figure 11:**
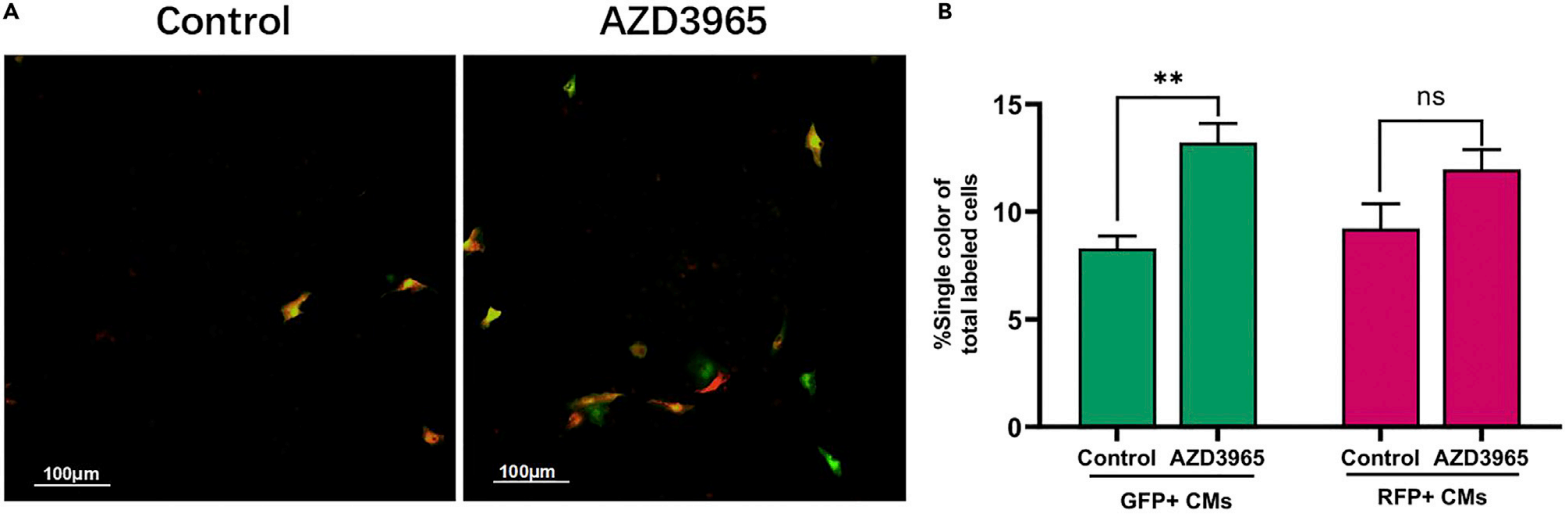
The compound AZD3965 slightly induces cardiomyocyte cytokinesis as assessed by the MADM reporter (A and B) Representative images (A) and quantification (B) of single-color (red or green) cells among Ad-Tnnt2-Cre adenovirus-infected neonatal mouse MADM cardiomyocytes after either DMSO or AZD3965 treatment. n = 2,500 cells per replicate, 3 replicates per group. Data are the mean ± SEM.; ∗∗p <0.01; ns, not significant; unpaired, two-tailed Student’s t test. Scale bars, 100 μm.
96. Calculate the percentage of single-color cardiomyocytes in each well. Troubleshooting 9.

## Expected outcomes

This protocol provides a method to discover novel small molecules promoting cardiomyocytes proliferation. An effective compound increases the percentage of mAG-hGeminin^+^ CMs (Figure 10) and single-color MADM CMs (Figure 11). The positive ratio calculated automatically by the software allows us to compare the ability of compounds on promoting cardiomyocyte proliferation as described.^1^

## Quantification and statistical analysis

### FUCCI analysis by MetaXpress

1. After imaging by MD ImageXpress Micro XL Imaging System, analyze the images in the custom module using MetaXpress software (Figure 12). Import the images and create the image names in different channels.

**Figure 12:**
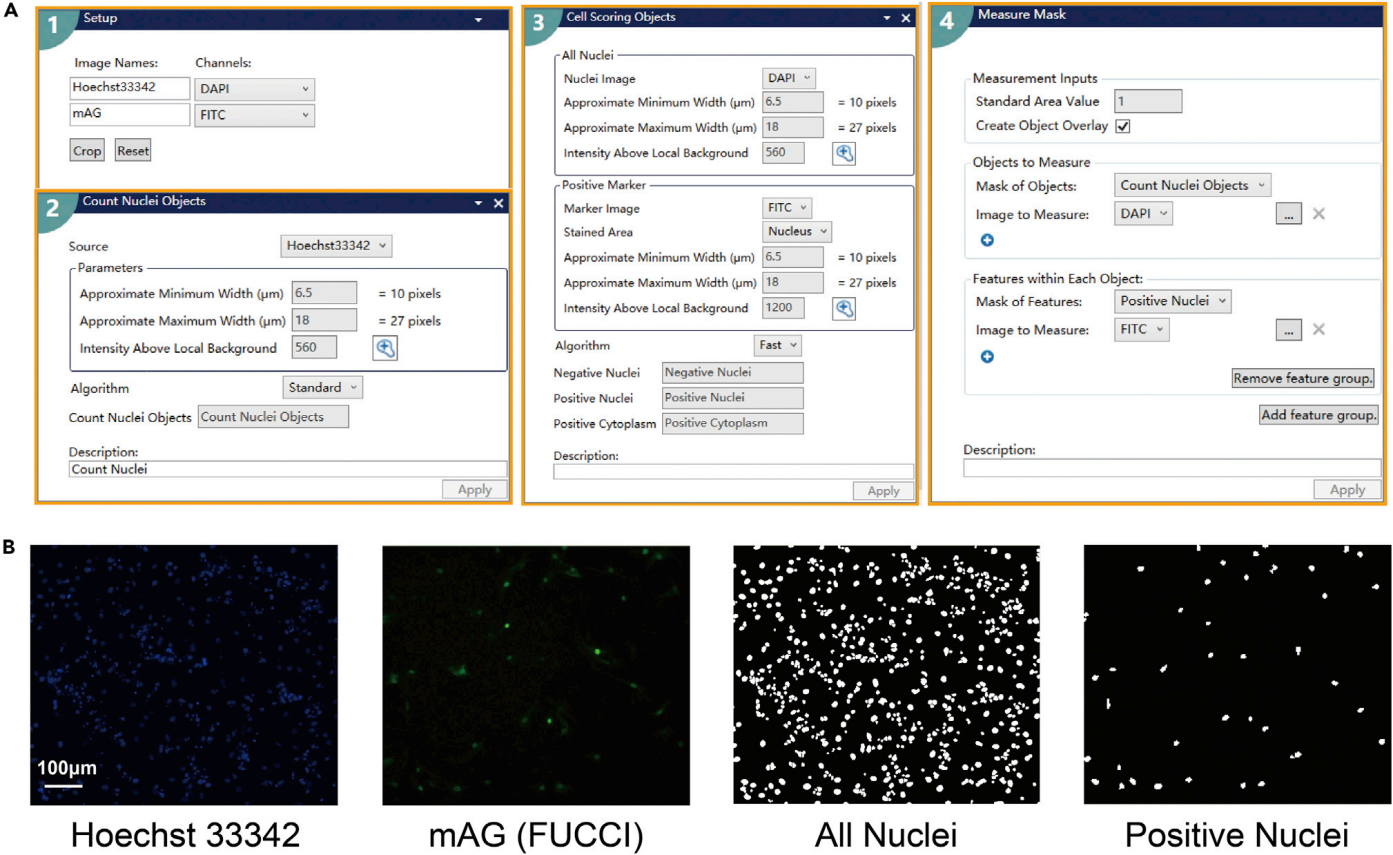
Diagram of analysis processes using MD MetaXpress software (A) Flow chart of analyzing the proportion of FUCCI-positive cardiomyocytes using MD MetaXpress software. (B) Representative images of Hoechst 33342, mAG, all nuclei, and FUCCI-positive nuclei. Scale bar, 100μm.
2. Use the count nuclei objects module in the software, set up the cell width and fluorescence intensity of Hoechst 33342 above the background to recognize nuclei.
3. Use the cell scoring objects module in the software, set up the threshold to the fluorescence intensity of mAG above the background to identify positive nuclei.
4. Export the total numbers of nuclei and positive nuclei.
5. Use Excel or other software to calculate the positive ratio.
  ***Optional:*** FUCCI analysis by harmony.
6. Alternatively analyze the images using Harmony Software (Figure 13). Use the image analysis module in Harmony to create a new analysis and then import the images. Identify nuclei by Hoechst 33342 staining. Identify single nuclei by adjusting parameters such as nuclear area, split factor, and fluorescence contrast.

**Figure 13:**
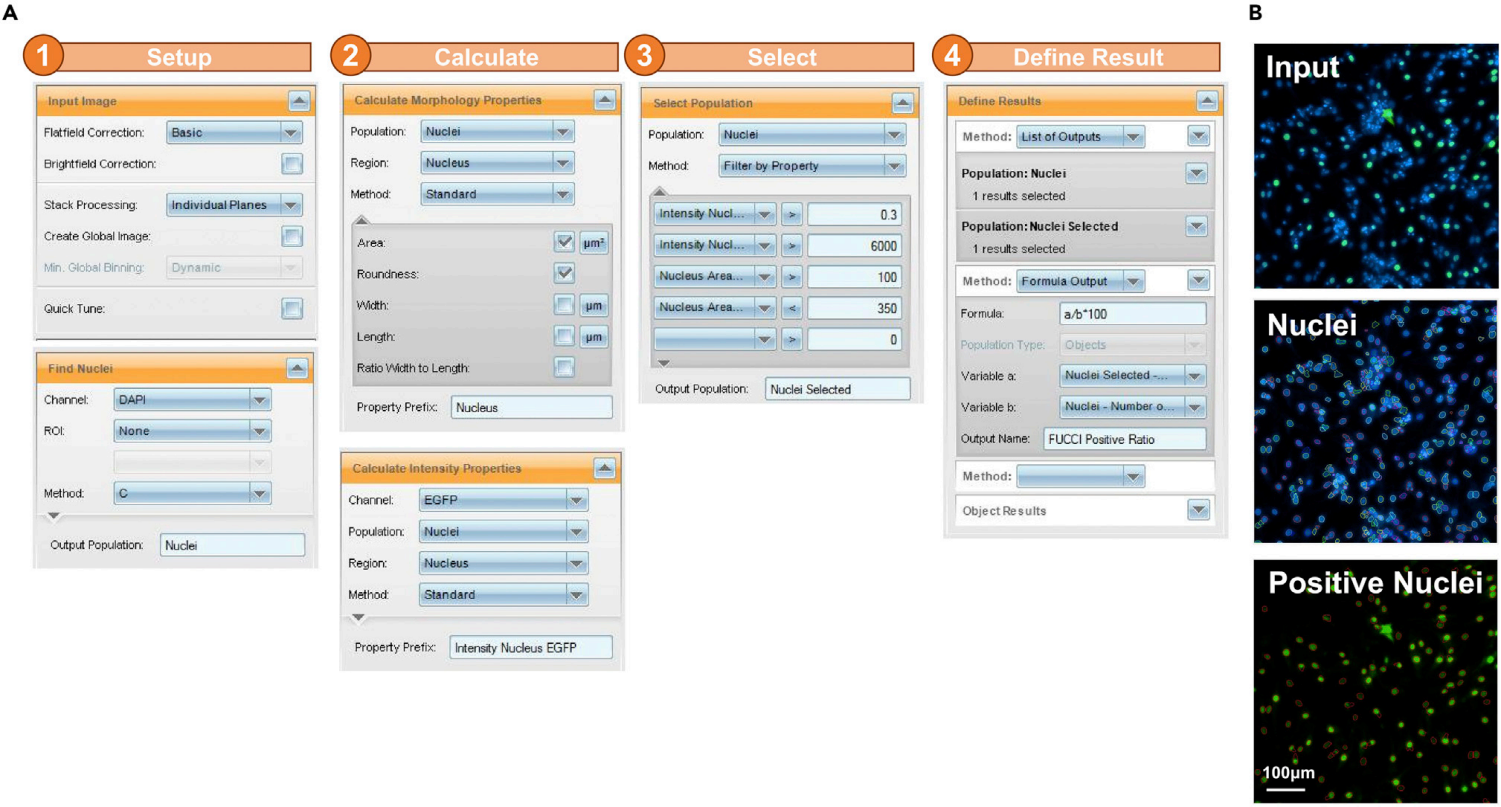
Diagram of analysis processes using Harmony software (A) Flow chart for analyzing the proportion of FUCCI-positive cardiomyocytes using Harmony software. (B) Representative input images, nuclei, and positive nuclei. Scale bar, 100μm.

***Note:*** To correct the vignetting effect of the images, the flatfield correction mode can be adjusted to basic or advanced.

7. Calculate morphological properties. To avoid identifying spurious signals or large cell clumps; nuclear areas that are not within the normal range should be removed.
8. Calculate intensity properties. Since mAG localizes with the nuclei, select positive cells by setting a threshold for the mean fluorescence intensity of mAG within the detected nuclei.

***Note:*** If positive cells cannot be accurately identified by the mean intensity alone, more thresholds can be set up by calculating contrast and maximum fluorescence intensity.

9. Select the population. Apply several thresholds to find the population that has a high fluorescence intensity.

***Note:*** Since the fluorescence intensity is related to the expression level of mAG and exposure time, it is necessary to reset thresholds in different experiments to select positive cells.

10. Define results. Set formula output: calculates the positive ratio [%] = (Number of selected nuclei / Total number of nuclei) × 100.

### MADM analysis

The analysis process for MADM cardiomyocytes is similar to the above method. However, when analyzing the number of single fluorescent cells, it is necessary to set a threshold to remove cells with fluorescence in another channel.

## Limitations

The limitation of this high-throughput chemical screening system is that the screening results may be affected by several factors, such as the quality of primary cardiomyocytes, the concentration of small molecules in the primary screening, and the treatment time with small molecules. Therefore, in order to ensure consistency and reliability of the screening results, it is necessary to ensure that cultured cardiomyocytes are in a good state, consider two additional repeating screens, and optimize the small molecule concentrations and treatment times. In addition, neonatal cardiomyocytes have some degree of proliferation that is different from adult cardiomyocytes, thus the outcome of this chemical screening needs to be further verified in adult cardiomyocytes. Furthermore, this method can be used for any cell types; however, current protocol is using primary neonatal rat or mouse cardiomyocytes for the screening and characterization of the compounds. In general, any cell-specific promoters can be used for driving FUCCI and MADM reporter expression, and thus, these reporters can be used for performing chemical screening on any types of cells. Due to the depth of field (DOF) of the objectives we used in MD and PE high-content microscopy will not be able to distinguish different layers of cells like the fibroblasts and cardiomyocytes, it is necessary to exclude the possibility of compounds acting on other cells contaminated including fibroblast cells by immunostaining. We infected FUCCI and MADM viruses with cardiomyocytes or fibroblasts, and found that these fluorescent reporters were specifically expressed in cardiomyocytes but not in fibroblasts. Therefore, it’s necessary to confirm the specificity of reporter gene expression when applying the FUCCI and MADM systems to other cells.

## Troubleshooting

### Problem 1

Tissues stick together during heart digestion, resulting in fewer viable cardiomyocytes *(step 15*).

### Potential solution

This may be due to DNA released from lysed cells when they die, making the tissues stick together. Do not slice the heart into too small pieces and reduce the stir speed during digestion. Besides, an appropriate amount of DNase can be added to decrease tissue clumping.

### Problem 2

Some cardiomyocytes do not adhere and are suspended in the culture medium *(step 31)*.

### Potential solution

• Optimize digestion conditions.
• Pretreat the plates with L-poly-lysine or laminin.
• Do not move plates within 48 h of cell seeding.

### Problem 3

No fluorescence signal after adenovirus infection *(step 34)*.

### Potential solution

• Pipette the dissolved adenovirus up and down to mix it well.
• Try a new aliquot of adenovirus.
• This may be caused by less efficient adenoviral infection, and you may increase the virus titer or increase infection time up to 48 h.

### Problem 4

**Figure 14:**
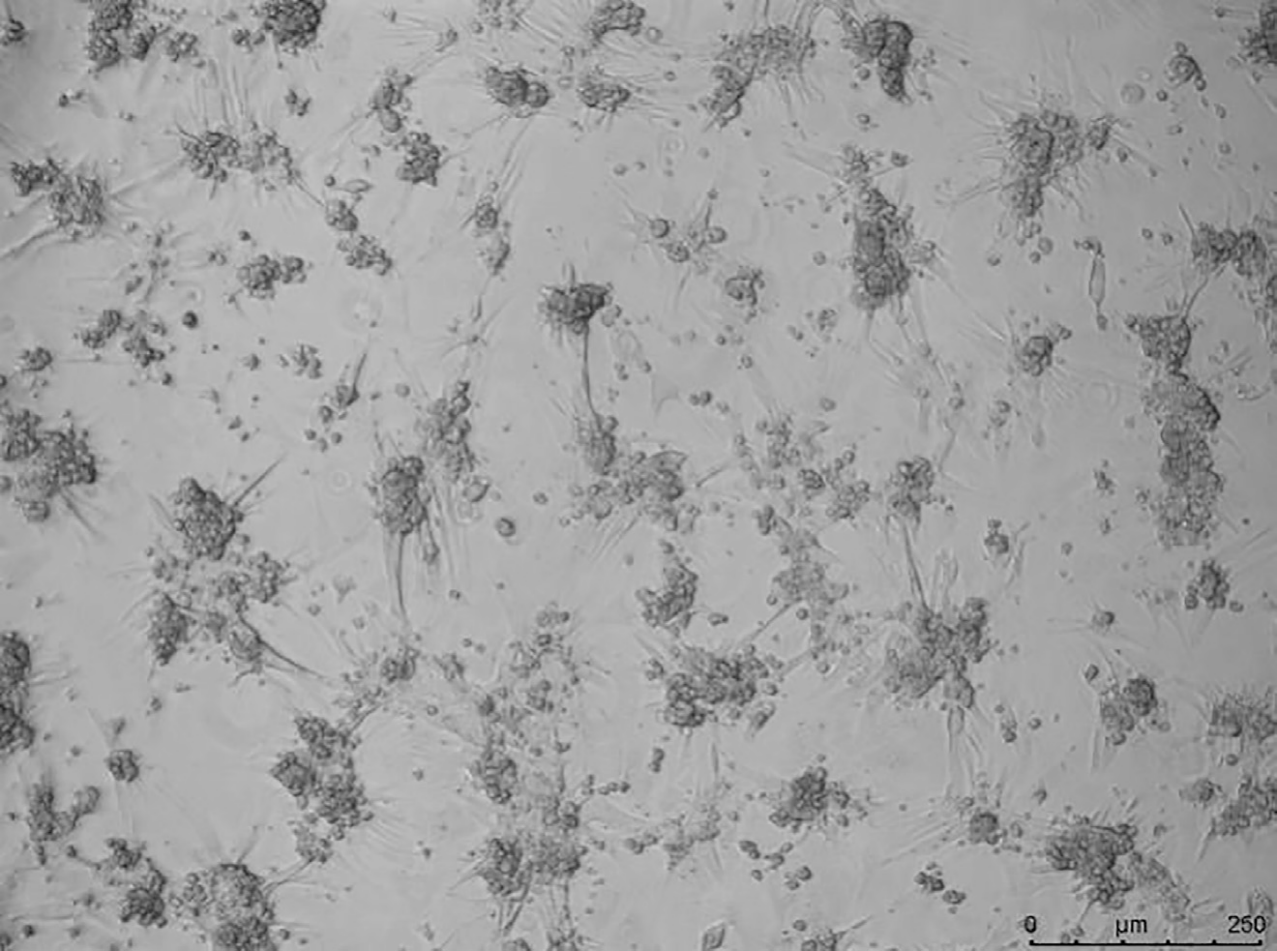
Cardiomyocytes aggregate during adherent culture. Scale bar, 250μm.

Cardiomyocytes aggregate during adherent culture (Figure 14) *(step 34)*.

### Potential solution

• This may be caused by poor quality of cardiomyocytes. Avoid over-digestion and possible mechanical damage to cardiomyocytes during digestion and plating.
• Excessive cell density may also cause aggregation during culture.
• Decrease the FUCCI virus titer.

### Problem 5

Few adhered live cardiomyocytes in the middle of the well in 96-well plates *(step 45)*.

### Potential solution

This may be caused by the strong force of ultrasound or the toxicity of certain compounds to cultured cardiomyocytes during drug deployment. Dead cardiomyocytes in the middle field of the well should not be imaged and analyzed.

### Problem 6

The percentage of FUCCI-positive cardiomyocytes in the positive control or negative control groups is too low *(step 48)*.

### Potential solution

• This could be caused by poor quality of cultured cardiomyocytes, and so repeat the experiments by re-isolating neonatal rat or mouse cardiomyocytes.
• Try a new aliquot of FBS or increase the percentage of FBS in the medium.
• Increase the FUCCI virus titer.

### Problem 7

Drug screening results are not reproducible *(step 49)*.

### Potential solution

• This may be caused by poor quality of cultured cardiomyocytes, and so repeat the experiments by re-isolating neonatal rat or mouse cardiomyocytes.
• Check the storage conditions and lifetime of the chemical library, and repeat screening with new aliquots of compounds or order new compounds.

### Problem 8

The percentage of FUCCI-positive cardiomyocytes is higher in the edge wells of 96-well plates *(step 54)*.

### Potential solution

This may be caused by the edge effect. Exclude data from edge wells during analysis, and small molecules arranged at the edge wells of 96-well plates should be rearranged to the middle wells of the plate for repeated screening.

### Problem 9

Poor repeatability between replicates in the same 96-well plate *(steps 54 and 96)*.

### Potential solution

• Mix cardiomyocytes well before plating by pipetting up and down to ensure that the cell density is similar between replicates.
• Fully mix the compounds before adding them to cardiomyocytes.

## Resource availability

### Lead contact

Further information and requests for resources and reagents should be directed to and will be fulfilled by the Lead Contact Dr. Jing-Wei Xiong (jingwei_xiong@pku.edu.cn).

### Materials availability

Tnnt2-mAG-hGeminin (1/110) and Tnnt2-Cre plasmids described in this study are available upon request.

## Data Availability

This study did not generate any datasets or code.

## Supplemental information

Data S1. Tnnt2 promoter sequences, mAG-hGeminin(1/110) coding sequences, and Cre coding sequences, related to steps 3 and 36
