# Supplementary material for: Protocol to identify small molecules promoting rat and mouse cardiomyocyte proliferation based on the FUCCI and MADM reporters
Source: STAR Protoc. 2022 Dec 5;3(4):101903. doi: 10.1016/j.xpro.2022.101903 (PMC9732407; doi:10.1016/j.xpro.2022.101903)
Supplement: Data S1. Tnnt2 promoter sequences, mAG-hGeminin(1/110) coding sequences, and Cre coding sequences, related to steps 3 and 36 [file mmc1.pdf]

Data S1.Tnnt2 promoter sequences, mAG-hGeminin(1/110) coding sequences, and Cre coding sequences, related to step 3 and 36

Tnnt2 promoter:

TGTAGTTAAT GATTAACCCG CCATGCTACT TATCTACCAG

GGTAATGGGG ATCCTCTAGA ACTATAGCTA GAATTCGCCC

TTACGGGCCC CCCCTCGAGG TCGGGATAAA AGCAGTCTGG

GCTTTCACAT GACAGCATCT

GGGGCTGCGG CAGAGGGTCG GGTCCGAAGC GCTGCCTTAT

CAGCGTCCCC AGCCCTGGGA GGTGACAGCT GGCTGGCTTG

TGTCAGCCCC TCGGGCACTC

ACGTATCTCC GTCCGACGGG TTTAAAATAG CAAAACTCTG

AGGCCACACA ATAGCTTGGG CTTATATGGG CTCCTGTGGG

GGAAGGGGGA GCACGGAGGG

GGCCGGGGCC GCTGCTGCCA AAATAGCAGC TCACAAGTGT

TGCATTCCTC TCTGGGCGCC GGGCACATTC CTGCTGGCTC

TGCCCGCCCC GGGGTGGGCG

CCGGGGGGAC CTTAAAGCCT CTGCCCCCCA AGGAGCCCTT

CCCAGACAGC CGCCGGCACC CACCGCTCCG TGGGACGATC

CCCGAAGCTC TAGAGCTTTA

TTGCGGTAGT TTATCACAGT TAAATTGCTA ACGCAGTCAG

TGCTTCTGAC ACAACAGTCT CGAACTTAAG CTGCAGAAGT

TGGTCGTGAG GCACTGGGCA

GGTAAGTATC AAGGTTACAA GACAGGTTTA AGGAGACCAA

TAGAAACTGG GCTTGTCGAG ACAGAGAAGA CTCTTGCGTT TCTGATAGGC ACCTATTGGT

CTTACTGACA TCCACTTTGC CTTTCTCTCC ACAGGTGTCC ACTCCCAGTT CAATTACAGC TCTTAAGGCT AGAGTACTTA ATACGACTCA CTATAG

mAG-hGeminin(1/110) coding sequences:

ATGGTGAG CGTGATCAAG CCCGAGATGA AGATCAAGCT GTGCATGAGG GGCACCGTGA ACGGCCACAA CTTCGTGATC GAGGGCGAGG GCAAGGGCAA

CCCCTACGAG GGCACCCAGA TCCTGGACCT GAACGTGACC GAGGGCGCCC CCCTGCCCTT CGCCTACGAC ATCCTGACCA CCGTGTTCCA GTACGGCAAC

AGGGCCTTCA CCAAGTACCC CGCCGACATC CAGGACTACT TCAAGCAGAC CTTCCCCGAG GGCTACCACT GGGAGAGGAG CATGACCTAC GAGGACCAGG

GCATCTGCAC CGCCACCAGC AACATCAGCA TGAGGGGCGA CTGCTTCTTC TACGACATCA GGTTCGACGG CACCAACTTC CCCCCCAACG GCCCCGTGAT

GCAGAAGAAG ACCCTGAAGT GGGAGCCCAG CACCGAGAAG ATGTACGTGG AGGACGGCGT GCTGAAGGGC GACGTGAACA TGAGGCTGCT GCTGGAGGGC

GGCGGCCACT ACAGGTGCGA CTTCAAGACC ACCTACAAGG CCAAGAAGGA GGTGAGGCTG CCCGACGCCC ACAAGATCGA CCACAGGATC GAGATCCTGA

AGCACGACAA GGACTACAAC AAGGTGAAGC TGTACGAGAA CGCCGTGGCC AGGTACTCCA TGCTGCCCAG CCAGGCCAAG GGATATCCAT CACACTGGCG

GCCGCTCGAG ATGAATCCCA GTATGAAGCA GAAACAAGAA GAAATCAAAG AGAATATAAA GAATAGTTCT GTCCCAAGAA GAACTCTGAA GATGATTCAG

CCTTCTGCAT CTGGATCTCT TGTTGGAAGA GAAAATGAGC TGTCCGCAGG CTTGTCCAAA AGGAAACATC GGAATGACCA CTTAACATCT ACAACTTCCA

GCCCTGGGGT TATTGTCCCA GAATCTAGTG AAAATAAAAA TCTTGGAGGA GTCACCCAGG AGTCATTTGA TCTTATGATT AAAGAAAATC CATCCTCTCA

GTATTGGAAG GAAGTGGCAG AAAAACGGAG AAAGGCGCTG

Cre coding sequences:

ATGGCCAA TTTACTGACC GTACACCAAA ATTTGCCTGC ATTACCGGTC GATGCAACGA GTGATGAGGT TCGCAAGAAC CTGATGGACA TGTTCAGGGA

TCGCCAGGCG TTTTCTGAGC ATACCTGGAA AATGCTTCTG

TCCGTTTGCC GGTCGTGGGC GGCATGGTGC AAGTTGAATA

ACCGGAAATG GTTTCCCGCA

GAACCTGAAG ATGTTCGCGA TTATCTTCTA TATCTTCAGG

CGCGCGGTCT GGCAGTAAAA ACTATCCAGC AACATTTGGG

CCAGCTAAAC ATGCTTCATC

GTCGGTCCGG GCTGCCACGA CCAAGTGACA GCAATGCTGT

TTCACTGGTT ATGCGGCGGA TCCGAAAAGA AAACGTTGAT

GCCGGTGAAC GTGCAAAACA

GGCTCTAGCG TTCGAACGCA CTGATTTCGA CCAGGTTCGT

TCACTCATGG AAAATAGCGA TCGCTGCCAG GATATACGTA

ATCTGGCATT TCTGGGGATT

GCTTATAACA CCCTGTTACG TATAGCCGAA ATTGCCAGGA

TCAGGGTTAA AGATATCTCA CGTACTGACG GTGGGAGAAT

GTTAATCCAT ATTGGCAGAA

CGAAAACGCT GGTTAGCACC GCAGGTGTAG AGAAGGCACT

TAGCCTGGGG GTAACTAAAC TGGTCGAGCG ATGGATTTCC

GTCTCTGGTG TAGCTGATGA

TCCGAATAAC TACCTGTTTT GCCGGGTCAG AAAAAATGGT

GTTGCCGCGC CATCTGCCAC CAGCCAGCTA TCAACTCGCG

CCCTGGAAGG GATTTTTGAA

GCAACTCATC GATTGATTTA CGGCGCTAAG GATGACTCTG

GTCAGAGATA CCTGGCCTGG TCTGGACACA GTGCCCGTGT

CGGAGCCGCG CGAGATATGG

CCCGCGCTGG AGTTTCAATA CCGGAGATCA TGCAAGCTGG

TGGCTGGACC AATGTAAATA TTGTCATGAA CTATATCCGT

AACCTGGATA GTGAAACAGG

GGCAATGGTG CGCCTGCTGG AAGATGGCGA TTAG
